# VLM-Assisted Routing and HU-Traceable DICOM Adaptation for Lumbar CT HU Measurement: A Deployment-Oriented Pilot Technical Evaluation

**DOI:** 10.3390/jimaging12080385

**Published:** 2026-08-14

**Authors:** Zhe-Yu Ye, Jun-Mu Peng, Tamotsu Kamishima

**Affiliations:** Faculty of Health Sciences, Hokkaido University, Sapporo 060-0812, Japan

**Keywords:** opportunistic CT, Hounsfield units, lumbar spine, vision-language model, DICOM, field of view, orientation normalization, quality control, technical evaluation

## Abstract

Automated lumbar computed tomography (CT) Hounsfield unit (HU) measurement can support opportunistic osteoporosis screening, but heterogeneous Digital Imaging and Communications in Medicine (DICOM) inputs often disrupt automated workflows before measurement. We evaluated a local Qwen2.5-VL-7B vision-language model (VLM)-assisted front end for suitability routing, input planning, and HU-traceable DICOM-derived field-of-view/orientation adaptation upstream of an unchanged single-slice lumbar CT HU workflow. The VLM was restricted to routing and planning. In a 20-case FUJIFILM pilot cohort, observed HU output availability was higher with the front-end-assisted route than with direct processing (12/20 versus 5/20), while paired automatic-success cases showed excellent agreement with post hoc manual ImageJ (version 1.54g) measurements (ICC(A,1) = 0.997; MAE = 4.64 HU). Public DICOM stress testing further demonstrated improved workflow robustness across heterogeneous datasets. These findings support the use of a deployment-oriented front-end strategy to improve the auditability and robustness of automated lumbar CT HU measurement while preserving an unchanged downstream measurement workflow.

## 1. Introduction

Osteoporosis is a major public health problem in aging societies because low bone strength increases fragility fracture risk, loss of independence, and healthcare burden. Nevertheless, many patients at risk remain undiagnosed or unscreened, in part because dedicated bone mineral density testing is not performed for every patient who undergoes routine imaging for other clinical indications. This gap has motivated interest in opportunistic imaging-based approaches that extract bone-health information from existing examinations rather than requiring a separate screening visit.

Routine CT is particularly attractive for opportunistic bone assessment because vertebral trabecular attenuation can be measured in Hounsfield units (HUs) from scans acquired for abdominal, oncologic, vascular, or other indications. Prior studies have shown that lumbar vertebral HU values correlate with bone mineral density and fracture risk and can provide practical surrogate markers of bone health [1,2,3,4,5,6,7,8]. For opportunistic screening to become scalable, however, HU measurement must be reproducible, auditable, and deployable across the heterogeneous CT data encountered in routine practice.

Automated and semi-automated vertebral/spine analysis workflows, including vertebral localization, segmentation, fracture detection, and HU measurement, have therefore attracted increasing attention [9,10,11,12,13]. Our previously published single-slice lumbar CT HU measurement workflow [14], Automated Single-Slice Lumbar QCT HU Value Measurement with Clinical Workflow, established a stepwise, clinically auditable process that includes input suitability assessment, bone segmentation, slice selection, trabecular region-of-interest (ROI) extraction, HU calculation, and quality-control evidence. Its main value is the preservation of a transparent downstream measurement path, in which final HU values are computed from Digital Imaging and Communications in Medicine (DICOM) pixel data after RescaleSlope/RescaleIntercept conversion rather than from screenshots or normalized preview images.

Real-world deployment introduces a different challenge from internal model validation. External CT series vary by manufacturer, scanner model, reconstruction kernel, slice thickness, anatomical coverage, field of view (FOV), matrix size, DICOM orientation, and display state. A workflow developed for relatively standardized axial, bone-window-like, vertebra-centered lumbar inputs may fail when exposed to broad abdominal series, thoracic-dominant acquisitions, off-center anatomy, inverted presentation, or small vertebral targets. These failures can occur before HU calculation, for example at suitability routing, crop/centering, whole-bone segmentation, slice ranking, trabecular segmentation, or ROI availability.

The resulting knowledge gap is deployment robustness. Prior work has largely focused on segmentation, localization, or HU estimation performance under controlled or curated inputs. Less attention has been given to how an automated HU workflow should decide whether a heterogeneous DICOM series should enter the lumbar measurement channel, how it should adapt FOV and orientation without breaking HU traceability, and how it should document failures in a way that remains reviewable. Public DICOM resources further highlight metadata and orientation heterogeneity that may be hidden when data are converted to simple image formats [15,16,17,18,19], and radiology AI deployment guidance emphasizes transparent failure modes, domain shift, and careful separation between technical performance and clinical claims [20,21,22,23,24,25,26,27].

This study evaluates a local vision-language model (VLM)-assisted front-end routing and HU-traceable DICOM-derived adaptation layer placed upstream of the unchanged downstream HU measurement workflow. Multimodal foundation models can interpret image appearance and metadata [28,29,30], but in this study the VLM was constrained to suitability routing, input planning, manual-review flagging, and FOV/orientation adaptation guidance. The objective was to test whether this frozen front-end strategy could improve deployability under heterogeneous DICOM inputs while preserving deterministic downstream HU measurement and auditable quality control. The work is framed as a frozen-adapter pilot external technical evaluation and public DICOM stress test, not as a diagnostic VLM study, bone-disease outcome validation, or definitive clinical-effectiveness study.

## 2. Materials and Methods

### 2.1. Study Design, Data Roles, and Endpoint Hierarchy

This study was designed as a pilot technical evaluation of deployment-related robustness rather than as a definitive clinical deployment-validation or downstream model-development study. The baseline route sent heterogeneous CT inputs directly into the original HU workflow. The proposed route first applied a local VLM-assisted front end for suitability routing and HU-traceable DICOM-derived FOV/orientation adaptation, then passed accepted or reviewable adapted series to the unchanged downstream HU measurement workflow. The primary technical endpoint was HU output availability in the Fuji20 pilot cohort. The secondary quantitative endpoint was automatic-versus-manual HU agreement in paired automatic-success Fuji20 cases. Public stress-test endpoints included HU availability, reviewability, and auditable failure evidence in Spine-Mets and PANCREAS-CT. Exploratory and supportive endpoints included appropriate exclusion, deterministic crop controls, focused reviewer adjudication, backward compatibility, and synthetic perturbation robustness. These endpoint layers were defined to avoid treating numeric HU availability as clinical correctness. The methodological roles and leakage controls of the datasets are summarized in Table 1.

The private external 20-case FUJIFILM pilot cohort (Fuji20) consisted of 20 Fuji CT cases. These cases were not used to train or fine-tune the VLM or the original workflow models. Early inspection of two representative cases clarified the prior HU workflow input requirement, namely an axial bone-window-like, vertebra-centered DICOM input rather than a broad full-body field of view. These two cases remained in the 20-case descriptive pilot analysis, but no case-specific thresholds, model weights, or parameters were adjusted. Fuji20 was therefore treated as the primary frozen-adapter pilot technical cohort with limited early input-requirement inspection, not as an untouched external validation cohort; its small sample size precludes definitive clinical-effectiveness claims.

Two public DICOM cohorts from The Cancer Imaging Archive (TCIA) were used as public stress-test and ablation evidence rather than outcome-validation cohorts. Spine-Mets-CT-SEG was queried through the National Biomedical Imaging Archive (NBIA) API; under the available NBIA/API access and download conditions, 26 of 55 metadata-listed CT series were successfully downloaded, including 16 GE and 10 Siemens series [16,17,18]. Because Spine-Mets is a spine-metastasis and radiotherapy-oriented collection, it was used for public DICOM workflow stress testing rather than bone-disease outcome validation. PANCREAS-CT was downloaded in full (80 DICOM series) and used as a non-target public stress-test cohort for suitability routing, orientation ensembling, spine localization, and over-acceptance analysis. PANCREAS-CT informed orientation engineering and is not treated as an untouched external clinical cohort.

The original selected-DICOM test input folder was evaluated only as a backward-compatibility control after the front-end adapter logic had been fixed. This selected-DICOM test set contained 47 already standardized prior-workflow-compatible inputs. It was not used for local VLM prompt engineering, crop-adapter development, routing-threshold selection, or model-weight tuning. The original workflow development/training data were not used to tune the local VLM-assisted front end. This separation was intended to test whether the proposed front end could preserve performance on inputs for which the original HU workflow was already appropriate, rather than to optimize the front end on the earlier development or test data.

Available DICOM header metadata were summarized to describe cohort heterogeneity without loading pixel data. Fuji20 consisted of FUJIFILM Healthcare Corporation/Supria CT series with 120 kVp acquisition, 5 mm slice thickness, and 512 × 512 matrices. Spine-Mets contained GE and Siemens CT series with variable slice thickness and reconstruction kernels. In the available PANCREAS-CT DICOM headers, matrix size and pixel spacing were available, whereas manufacturer, scanner model, tube voltage, and slice thickness fields were not reliably populated. These metadata summaries are reported as extracted rather than inferred.

### 2.2. Frozen Downstream HU Workflow and Rationale

The downstream HU workflow was deliberately kept frozen rather than retrained or fine-tuned. Retraining would change the boundary of the previously published auditable measurement workflow and would convert this study from a deployment-adapter evaluation into a new model-development study. It would also require a different annotation program, including systematic vertebral-level localization, lumbar-coverage labels, suitability labels, segmentation references, and ROI references across heterogeneous CT protocols. More importantly, routing is not identical to segmentation generalization: even a stronger downstream segmentation model would still need to determine whether a given DICOM series should enter a lumbar HU measurement channel. The present study therefore evaluates a detachable front-end planning layer. This design does not replace future retraining or fine-tuning studies; rather, it defines a conservative comparison point against which future downstream model adaptation can be evaluated.

The original HU workflow was kept unchanged [14]. It performs DICOM loading, HU conversion using RescaleSlope and RescaleIntercept, input classification, whole-bone segmentation, slice-quality ranking, trabecular segmentation, deterministic ROI construction, and HU summary generation. Candidate slices are ranked, the top 40% of ranked slices are retained as a candidate window, and slices require a rank score of at least 0.65 before trabecular segmentation and ROI/HU measurement. If no slice satisfies this downstream quality-control gate, the case is recorded as an auditable failure rather than forced into measurement.

Windowing parameters and preview images from the front end were used only for planning, visualization, and standardized presentation. Final HU values always came from the DICOM-derived HU array used by the unchanged downstream HU workflow.

### 2.3. VLM-Assisted Front-End Routing

A local Qwen2.5-VL-7B VLM was deployed as an off-the-shelf Ollama model package (qwen2.5vl:7b) through a local JavaScript Object Notation (JSON)-response inference interface on a workstation with an NVIDIA RTX 4090D GPU (NVIDIA Corporation, Santa Clara, CA, USA) [30]. No task-specific VLM weight training, fine-tuning, low-rank adaptation (LoRA) tuning, or case-level optimization was performed. For each DICOM series, metadata and a 3 × 3 montage sampled from the series were provided to the planner. The structured JSON output included axial CT status, spine/lumbar relevance, thoracic-dominant or lumbar-insufficient coverage flags, recommended display window parameters, target physical FOV, crop strategy, routing decision, quality flag, manual-review flag, confidence, and notes. In the executable pipeline, route decision and coverage flags determined whether the series was sent, held for review, or excluded; display-window fields supported preview generation and audit; and confidence/notes were retained for audit rather than used as fitted thresholds. The target-FOV and crop-strategy fields provided planning context, but the reported adapter used deterministic 180 mm series-fixed crop geometry and deterministic spine-candidate localization. The VLM calls used deterministic decoding settings that were supported by the runtime (temperature 0.0) and a 512-token response limit. The full prompt, JSON schema, inference setting summary, and JSON failure-handling notes are provided in the Supplementary Technical Appendix; the prompt instructed the VLM to output planning and routing fields only, not HU values, final segmentation, final ROI, or diagnostic conclusions. Because the front end combines VLM-based suitability routing with deterministic spine-localized adaptation and orientation normalization, this study evaluates the integrated front-end planning framework rather than isolating the VLM as a standalone performance source.

Because axial previews can be displayed upside down depending on DICOM geometry or viewer convention, an orientation-aware suitability reviewer was evaluated. A second 180-degree-rotated montage was reviewed, and the orientation ensemble routed a series to the downstream HU workflow if either presentation contained a credible lumbar or lower thoracolumbar candidate. This ensemble was used to reduce display-orientation sensitivity, not to create per-slice VLM crops.

### 2.4. Computational Environment and Runtime Documentation

The local VLM was run on a Windows workstation with an Intel Core i9-14900KF CPU (Intel Corporation, Santa Clara, CA, USA), an NVIDIA GeForce RTX 4090D GPU (NVIDIA Corporation, Santa Clara, CA, USA), 34.2 GB system RAM, and a local Ollama runtime. The local model package was qwen2.5vl:7b, reported by the runtime as an approximately 8.3-billion-parameter Qwen2.5-VL model with Q4_K_M quantization. Existing VLM-stage latency records were extracted from project output logs and summarized as runtime evidence. End-to-end timing was not prospectively recorded in the original experiments, and the archived full downstream environment was not reconstituted solely for this analysis. Therefore, the reported runtime results describe the local VLM inference stage rather than a complete end-to-end throughput benchmark. PyTorch and CUDA versions were not directly applicable because the VLM inference interface was managed by Ollama rather than directly through PyTorch or CUDA. The archived post-submission environment audit recorded Ollama version 0.31.2.

### 2.5. HU-Traceable DICOM-Derived FOV and Orientation Adaptation

Accepted or reviewable series underwent deterministic, HU-traceable DICOM-derived adaptation. The target visual style was an axial bone-window-like, spine-centered crop with a 180 mm physical FOV. The crop was series-fixed rather than slice-specific: a candidate spine center was estimated from sampled slices and then applied consistently across the selected range. DICOM orientation normalization was applied when ImageOrientationPatient indicated that the adapted pixel array would be opposite to prior-workflow-compatible axial geometry. Source DICOM files were never overwritten. The adapter copied and cropped original stored pixels while preserving RescaleSlope and RescaleIntercept, so final HU measurement remained DICOM-based.

Adapted DICOM copies were internal research derivatives used only to pass a standardized pixel presentation into the frozen downstream HU workflow. HU-relevant scaling tags were retained, while image-matrix fields and audit notes were updated for the cropped derivative. Detailed tag handling, unique identifier (UID) policy, ImagePositionPatient treatment, and transform-manifest fields are provided in the Supplementary Technical Appendix. Thus, “HU-traceable” refers to preserved stored-pixel and RescaleSlope/RescaleIntercept calibration for downstream HU-array calculation, not to standards-complete DICOM spatial interchange. The adapted copies were not Picture Archiving and Communication System (PACS)-ready diagnostic DICOM objects; a clinical interchange implementation should generate standards-complete derived objects with consistently updated patient-coordinate geometry.

**Figure 1:**
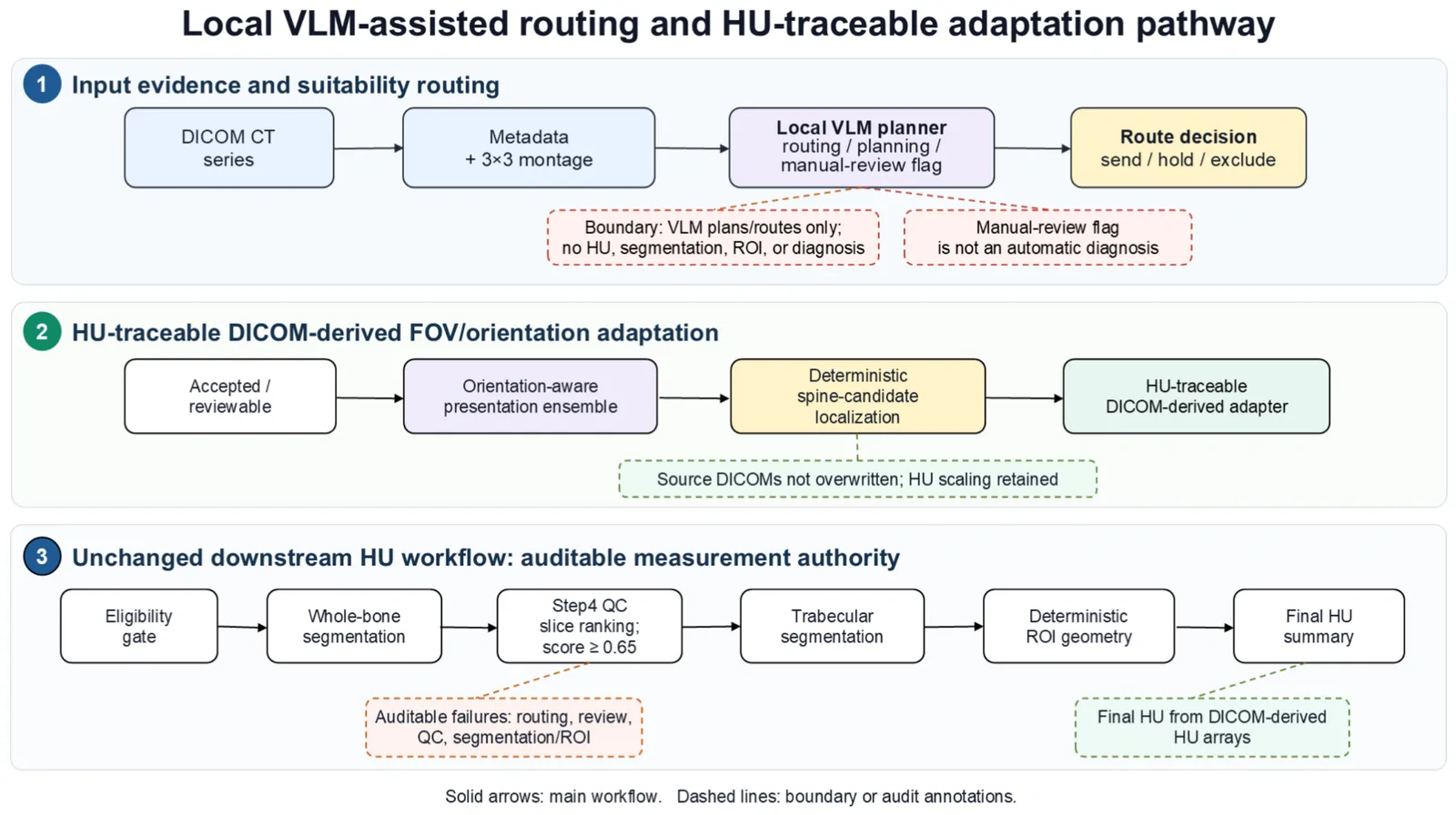
Overall local VLM-assisted suitability-routing and HU-traceable DICOM-derived adaptation workflow. The VLM reviews metadata and montage evidence for routing, input planning, and manual-review flags only. It does not measure HU, perform final vertebral segmentation, define the final ROI, or issue diagnoses. Final HU comes from the DICOM-derived HU array processed by the unchanged downstream HU workflow; Step 4 remains the downstream quality-control gate.

**Figure 2:**
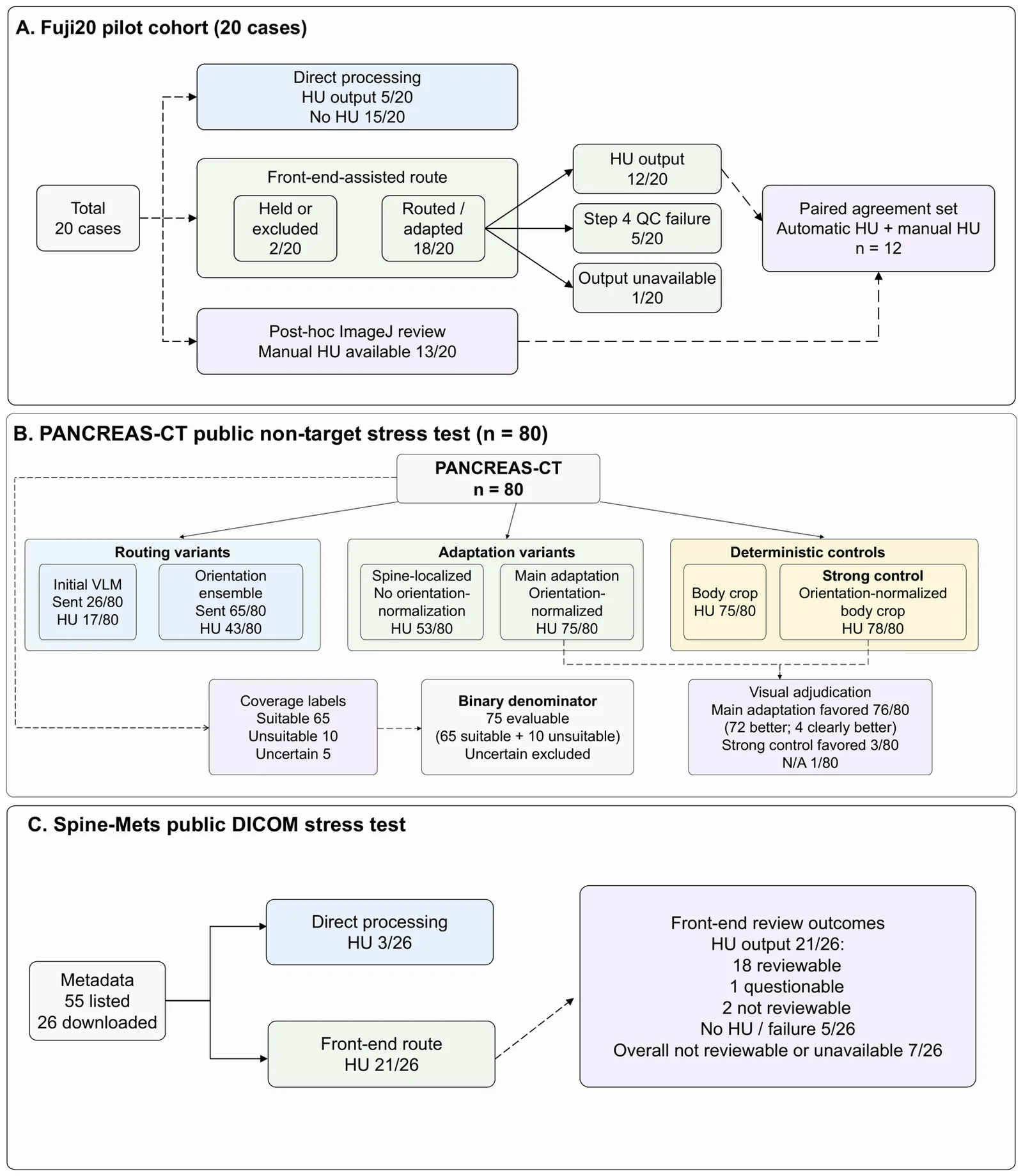

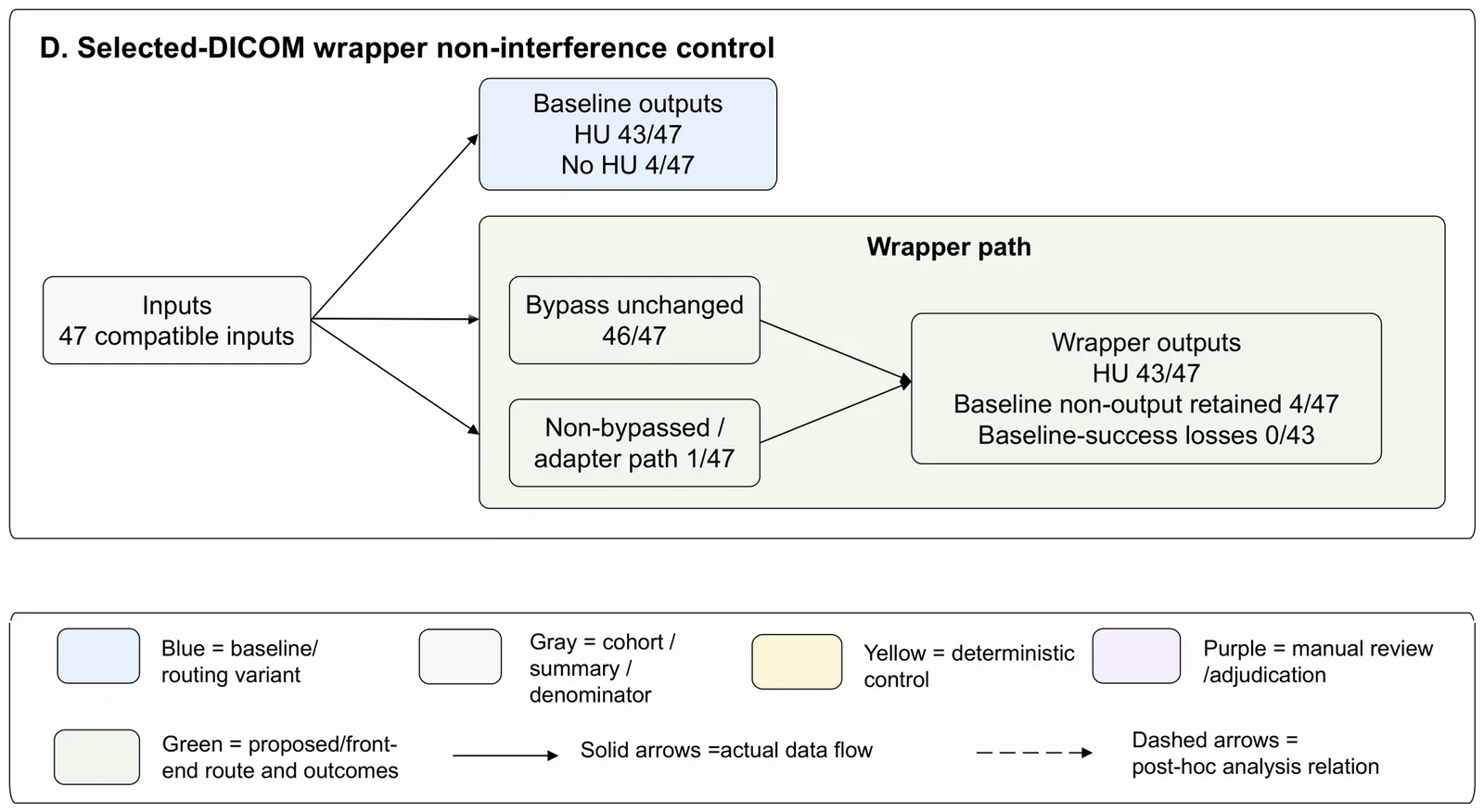
Cross-dataset cohort and parallel route overview. Panel (**A**) shows the Fuji20 pilot cohort. Branches represent alternative routes or analysis subsets rather than sequential attrition. Denominators are shown as cases or series as specified. Panel (**B**) shows PANCREAS-CT public non-target stress-test routes, adaptation variants, deterministic controls, post hoc coverage labels, and visual-adjudication subsets. Panel (**C**) shows the Spine-Mets public DICOM stress test and review outcomes. Panel (**D**) shows the selected-DICOM wrapper non-interference control; the legend defines color coding and arrow styles for all panels.

The overall front-end and downstream workflow is illustrated in Figure 1, and the parallel cohort and route structure is summarized in Figure 2.

Existing crop-transform records were summarized to address the physical-FOV concern. The 180 mm crop was analyzed as a downstream-compatibility FOV adaptation rather than a final vertebral bounding box. The transform summaries quantify crop width, crop area fraction, border-touching behavior, and rotation state where records were available; full-cohort vertebral mask occupancy, clipping rate, and margin metrics were not claimed where evaluated masks were unavailable.

A cross-dataset case-flow figure was created to show denominators for the pilot cohort, public stress-test cohorts, and compatibility control. Focused review subsets are shown as subsets of the relevant cohort rather than as whole-cohort validation endpoints.

### 2.6. Manual HU Evaluation and Statistical Analysis

Manual ImageJ HU review was performed as a post hoc quantitative sanity check for Fuji20 using a manual-review approach consistent with our previously published workflow [14,31]. A review package with case folders (001–020), mapping tables, original DICOM paths, automatic overlays, and selected-slice identifiers was generated for anatomical matching. Manual HU evaluation was performed by J.-M.P., a physician with six years of clinical experience in spine surgery and radiological image assessment. Manual HU values were recorded from the original DICOM images in ImageJ. Manual ROIs were placed in the trabecular vertebral body while avoiding the cortical shell, basivertebral venous plexus, focal sclerosis, osteophytes, metal artifacts, and visibly unsuitable anatomy. Multiple manual slices were summarized as a case-level mean HU. When exact one-to-one slice matching was not feasible, anatomically corresponding trabecular slices targeting the same vertebral region were used. Automatic overlays and selected-slice identifiers were used only for anatomical matching and case-level pairing, not for copying, adjusting, or validating the automatic ROI. Manual HU availability reflected completed and recorded post hoc review-package coverage, not workflow success, anatomical eligibility, or clinical suitability. Because this review was post hoc and not blinded, it was interpreted as a pilot quantitative sanity check rather than a reference-standard clinical agreement experiment. Manual HU values were not used to tune the VLM, adapter, or original HU workflow.

Availability was summarized as the proportion of cases with a final downstream HU workflow output. Wilson 95% confidence intervals were calculated for binary availability, reviewability, appropriate-exclusion, false-target, and adjudication proportions. Paired binary availability changes were assessed with exact McNemar tests; very small *p*-values are reported as p<0.001. Manual agreement was summarized using automatic-minus-manual bias, standard deviation of paired differences, mean absolute error (MAE), root mean square error (RMSE), Bland–Altman limits of agreement, Pearson correlation, and ICC(A,1) with bootstrap confidence interval [32,33]. Because paired manual HU analysis included only 12 automatic-success cases and therefore had automatic-success selection bias, it was interpreted as a pilot quantitative sanity check rather than definitive clinical agreement validation. No formal diagnostic-style sensitivity, specificity, precision, recall, F1 score, positive predictive value (PPV), or negative predictive value (NPV) analysis was performed against an independent blinded multi-reader reference standard. Exploratory PANCREAS-CT route-level sensitivity, specificity, PPV, NPV, accuracy, F1 score, false-acceptance rate, and false-exclusion rate were calculated only against single-reviewer, non-blinded binary coverage-based suitability labels.

### 2.7. Gate-Level VLM-On/Off Routing Analysis Design

A gate-level VLM-on/off routing-decision analysis was performed to separate semantic routing from geometric adaptation at the decision level. Direct raw-input processing was not considered a clean VLM-off comparison because it simultaneously removes routing, orientation normalization, spine localization, FOV adaptation, DICOM-derived adaptation, and standardized downstream presentation. The VLM-on route used the existing VLM orientation-ensemble routing decision. The VLM-off no-semantic pass-through route sent all technically eligible PANCREAS-CT series at the routing-decision level. A basic technical-integrity pass-through was also defined using only pre-VLM manifest/DICOM integrity fields, including valid case identifiers, series identifiers, image counts, and DICOM counts. The basic technical-integrity pass-through verified only minimal manifest and DICOM integrity and did not constitute a semantic or anatomical suitability router. Deterministic body-crop and orientation-normalized deterministic strong-control output policies were retained as high-sensitivity geometric controls, but were not treated as pure routing-only gates when defined by HU-output status.

The analysis used the existing single-reviewer PANCREAS coverage-based suitability labels. Positive labels indicated suitable lumbar or lower-thoracolumbar anatomical coverage, and negative labels indicated lumbar-insufficient or otherwise coverage-unsuitable status for the lumbar HU workflow. These labels did not constitute a complete clinical suitability reference for osteoporosis-related HU assessment, because they did not independently adjudicate all possible factors such as contrast phase, reconstruction kernel, metal, motion, focal bone lesions, fracture, or diagnostic threshold applicability. Five cases labeled as uncertain/manual review were excluded from the binary-metric denominator, leaving 75 evaluable cases. Metrics were reported with Wilson 95% confidence intervals for proportions and exact McNemar tests for paired route-disagreement and correctness-disagreement tables. Because the labels were single-reader, non-blinded, and post hoc, the analysis was interpreted as exploratory gate-level routing evidence rather than an independent clinical reference-standard validation. A fully rerun end-to-end matched pipeline comparison was not performed in this study. For the no-semantic and basic technical-integrity pass-through policies, the operational HU-output count was derived from archived all-series outputs generated by the orientation-normalized spine-localized route, which had previously been applied to all 80 PANCREAS-CT series. No new end-to-end rerun was performed for these gate-level policies. Deterministic body-crop and orientation-normalized deterministic strong-control HU-output counts were derived from archived geometric/control output records.

### 2.8. Controls, Compatibility Testing, and Reviewer Adjudication

A deterministic body-crop control was evaluated as a high-sensitivity geometric baseline. The initial version used deterministic body-center crop geometry without semantic suitability routing. A stronger control additionally used the same deterministic DICOM orientation normalization as the main adapter while retaining body-center crop geometry rather than spine-localized FOV planning. These controls tested whether geometric adaptation alone could recover HU outputs and prevented interpreting higher throughput as VLM/front-end superiority.

The original selected-DICOM compatibility analysis compared both availability and paired HU values. Already compatible selected-DICOM cases were expected to bypass front-end recropping and proceed directly to the unchanged original HU workflow. For bypassed cases, the wrapper did not create an adapted DICOM copy and did not invoke VLM-guided crop selection; it preserved the original prior-workflow-compatible input path. Absence of harm was assessed by no loss of baseline-success cases and no wrapper-induced change in paired HU values among cases with HU outputs in both routes. Paired HU equality in this control was interpreted as front-end non-interference, not as an independent repeatability experiment or as evidence that VLM preprocessing can reproduce HU exactly.

Focused reviewer adjudication was performed by the same physician reviewer (J.-M.P.) using predefined review tasks for the public conflict and stress-test sets. The review covered PANCREAS-CT VLM exclusions, PANCREAS-CT deterministic-crop conflicts, Spine-Mets public stress-test outputs, and a full non-blinded PANCREAS-CT comparison between the spine-localized orientation-normalized adaptation route, hereafter termed the main adaptation route, and the orientation-normalized deterministic body-crop strong-control. Cases were classified according to the predefined checklist; absence of adverse coverage, crop, ROI, or targeting findings was treated as reviewable status or appropriate exclusion, depending on the review task. The full PANCREAS comparison classified whether the main adaptation route or strong-control had better crop/segmentation/ROI quality and recorded the dominant strong-control issue when present. This review was used only as supportive visual evidence for centering, coverage, and reviewability trends, not as a formal superiority endpoint or blinded statistical reference standard. For exploratory PANCREAS-CT route-level metrics, positive labels were suitable lumbar or lower-thoracolumbar coverage and negative labels were lumbar-insufficient or unsuitable coverage; these should be interpreted as anatomical-coverage routing labels rather than complete clinical HU-measurement suitability labels. Five cases labeled as uncertain/manual review were excluded from the binary-metric denominator, leaving 75 evaluable cases. These metrics were interpreted as supportive deployment-routing evidence rather than definitive validation metrics.

## 3. Results

### 3.1. Primary Fuji20 Pilot Evaluation

**Figure 3:**
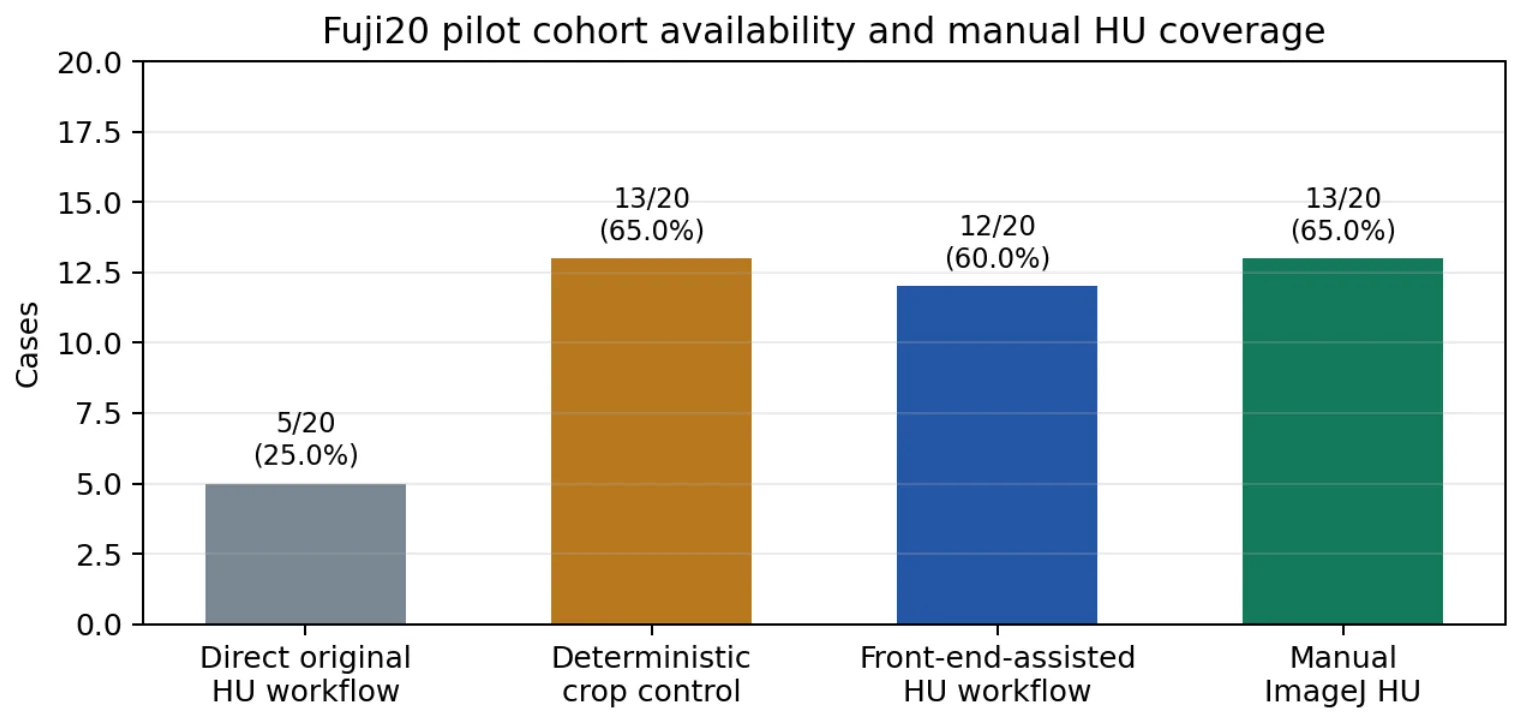
Fuji20 availability, deterministic crop control, and manual ImageJ HU coverage. Direct original-workflow processing produced 5/20 HU outputs, front-end-assisted prior-workflow processing produced 12/20 outputs, deterministic body crop produced 13/20 outputs, and manual ImageJ HU values were available for 13/20 cases. Manual ImageJ HU availability reflects post hoc review coverage, not workflow success, anatomical eligibility, or clinical suitability.

**Figure 4:**
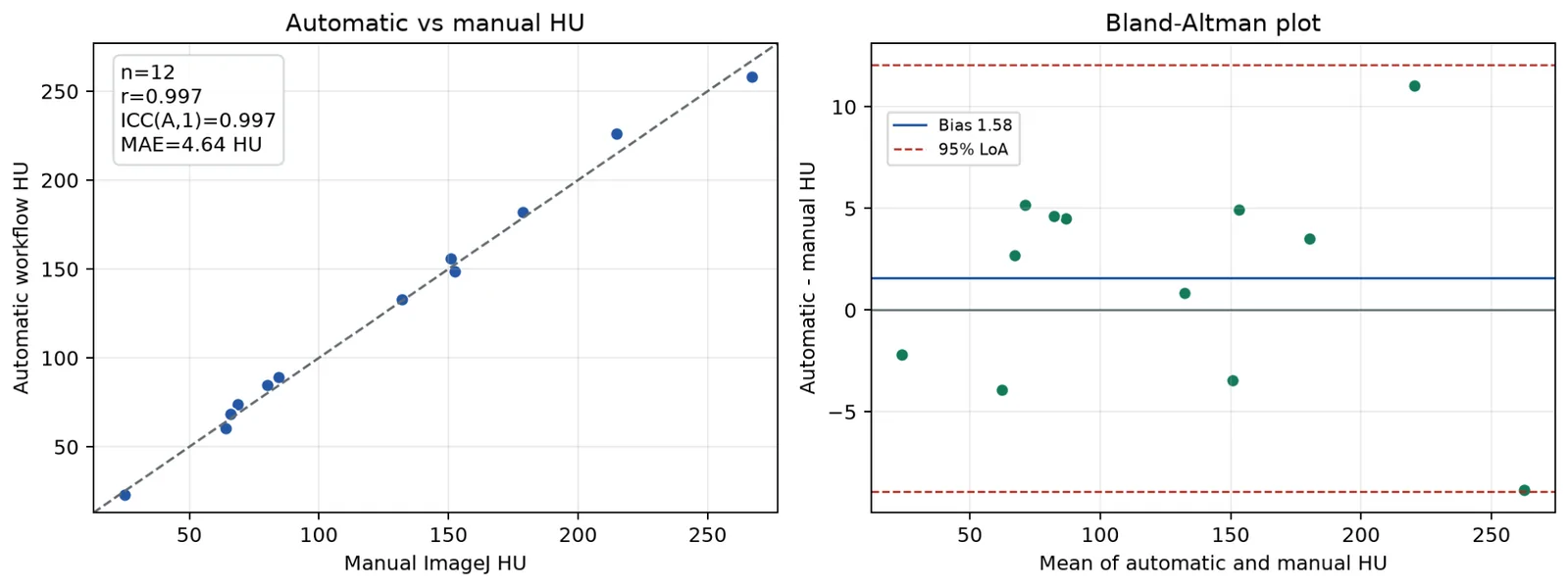
Automatic workflow HU versus manual ImageJ HU in paired Fuji20 automatic-success cases. (**Left**): scatter plot. (**Right**): Bland–Altman analysis. Manual review was a post hoc quantitative sanity check and not a blinded reference-standard experiment.

Fuji20 availability and manual HU coverage are summarized in Figure 3. Automatic-versus-manual HU agreement is shown in Figure 4. Case-level Fuji20 outcomes are provided in Supplementary Table S1. The manual ImageJ mapping is provided in Supplementary Table S2, and case-level failure evidence is provided in Supplementary Table S3.

In Fuji20, the direct original HU workflow produced HU outputs in 5/20 cases (25.0%; 95% CI, 11.2–46.9%), whereas the front-end-assisted route produced HU outputs in 12/20 cases (60.0%; 95% CI, 38.7–78.1%). In paired availability analysis, 3 cases produced HU outputs in both routes, 9 were front-end-only successes, 2 were baseline-only successes, and 6 failed in both routes; the exact two-sided McNemar *p*-value was 0.065. The deterministic body-crop control produced HU outputs in 13/20 cases (65.0%; 95% CI, 43.3–81.9%). The front-end-assisted route showed higher observed availability than direct original-workflow processing, although the paired difference did not reach conventional statistical significance (exact two-sided McNemar *p* = 0.065). Deterministic cropping recovered a similar number of outputs and should be interpreted as a strong geometric control rather than a weak baseline. The result supports the importance of FOV/orientation adaptation while preventing a claim that the VLM-assisted route is throughput-superior to deterministic cropping.

Manual ImageJ HU was available for 13/20 cases, including 12 paired automatic-success cases. Seven cases did not have recorded post hoc ImageJ HU values in the review package; these included five front-end/downstream non-output cases and two manual-review-held cases. This missingness was treated as incomplete post hoc review coverage and lack of paired agreement data, not as anatomical ineligibility or manual-measurement failure. In the paired subset, automatic and manual HU showed strong agreement: Pearson r=0.997, ICC(A,1) = 0.997 (95% CI, 0.994–0.998), automatic-minus-manual bias = +1.58 HU, SD of differences = 5.36 HU, Bland–Altman limits of agreement = −8.92 to +12.08 HU, MAE = 4.64 HU, RMSE = 5.37 HU, and maximum absolute error = 11.06 HU. Because the paired sample was small and restricted to automatic-success cases, this analysis was interpreted as a pilot quantitative sanity check rather than definitive clinical agreement validation.

In Fuji20, the front-end-assisted route showed higher observed HU output availability than direct original processing, while paired automatic-success cases showed close post hoc ImageJ agreement.

### 3.2. Public DICOM Stress Tests

**Figure 5:**
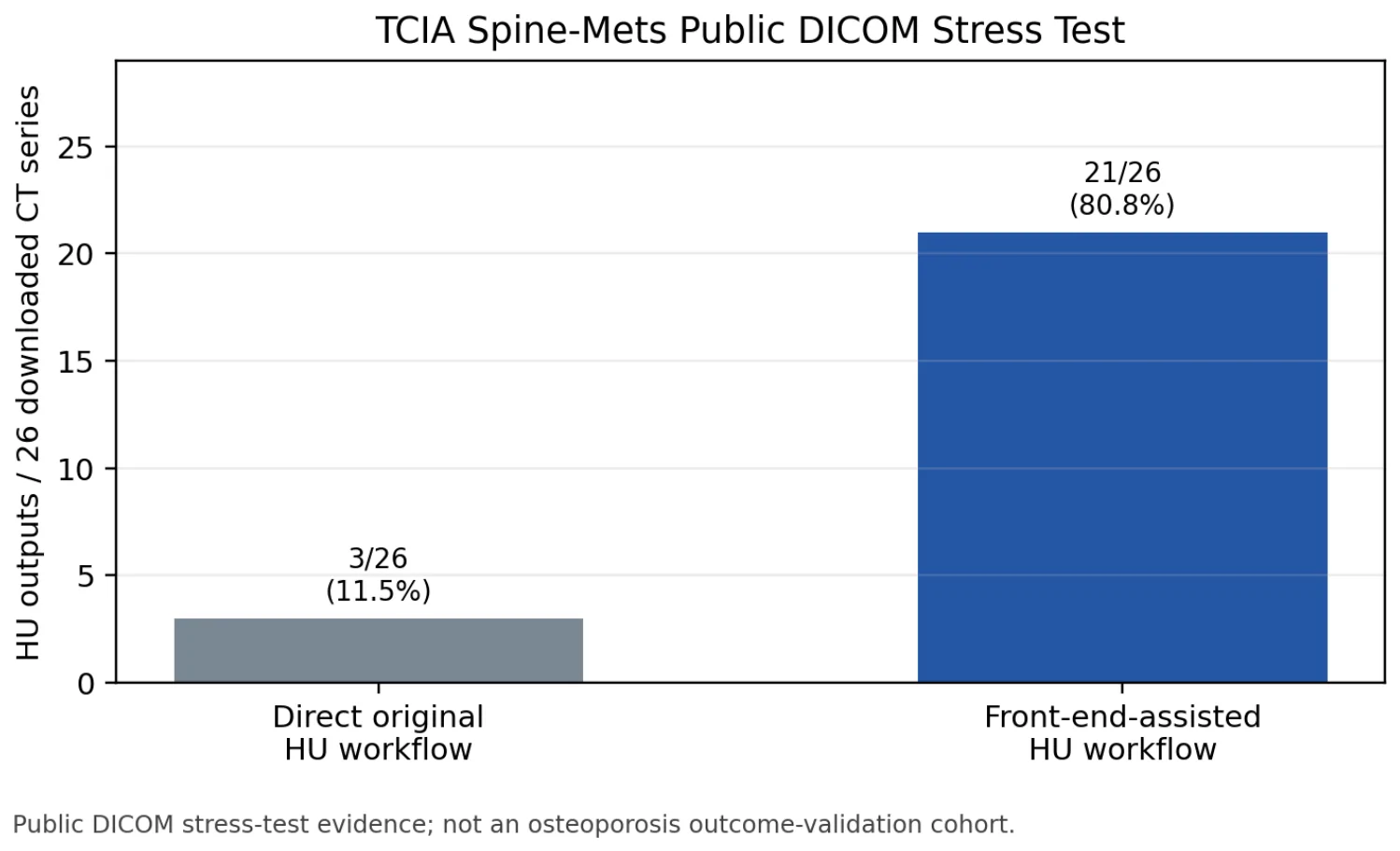
TCIA Spine-Mets public DICOM stress-test availability among the 26 successfully downloaded CT series. The cohort was used as a stress-test and engineering evidence rather than as a bone-disease outcome validation cohort.

**Figure 6:**
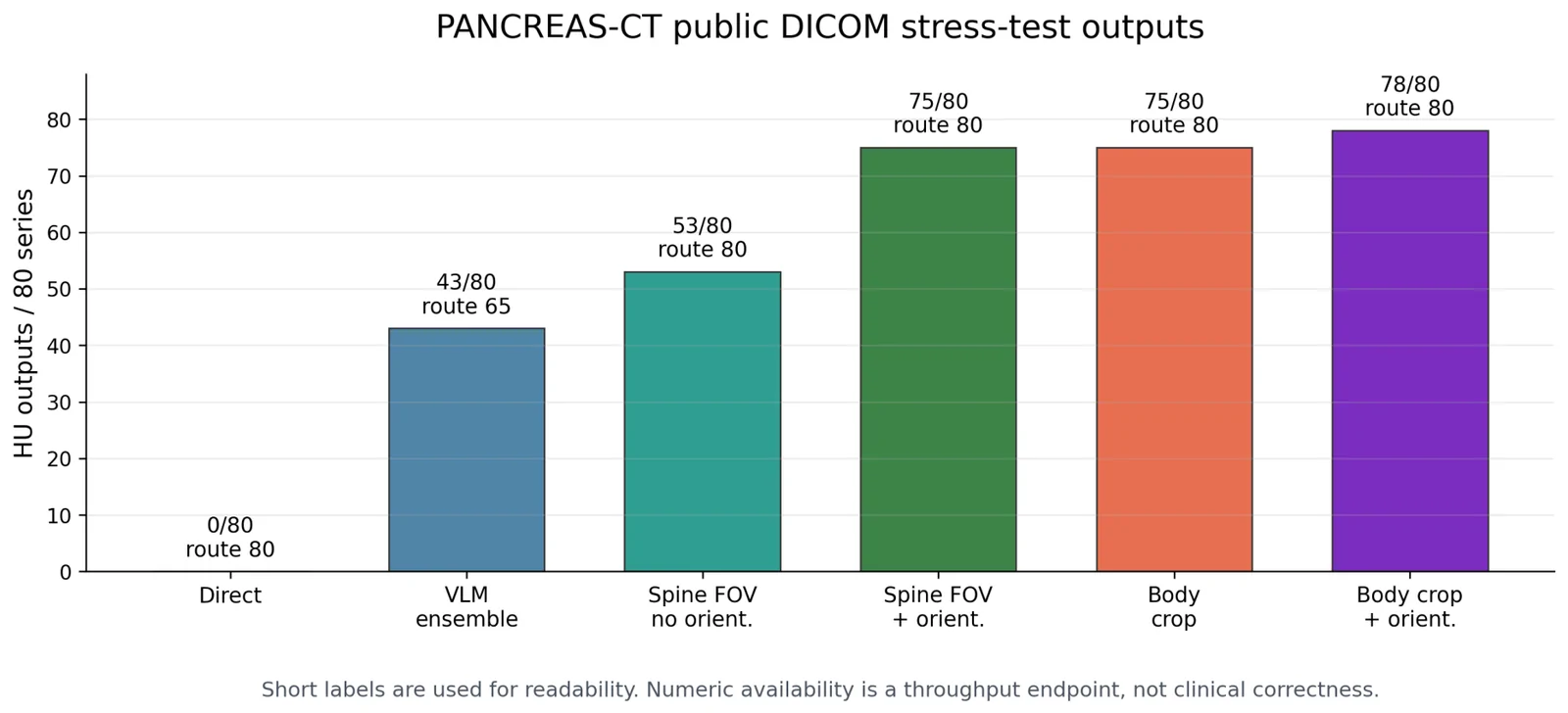
PANCREAS-CT public non-target stress-test strategy comparison. Direct original-workflow processing produced no HU outputs. Orientation-aware and spine-localized adaptation increased numeric availability, while the orientation-normalized deterministic body-crop strong-control demonstrates that high availability alone is not a sufficient marker of appropriate lumbar HU routing or reviewable measurement quality.

**Figure 7:**
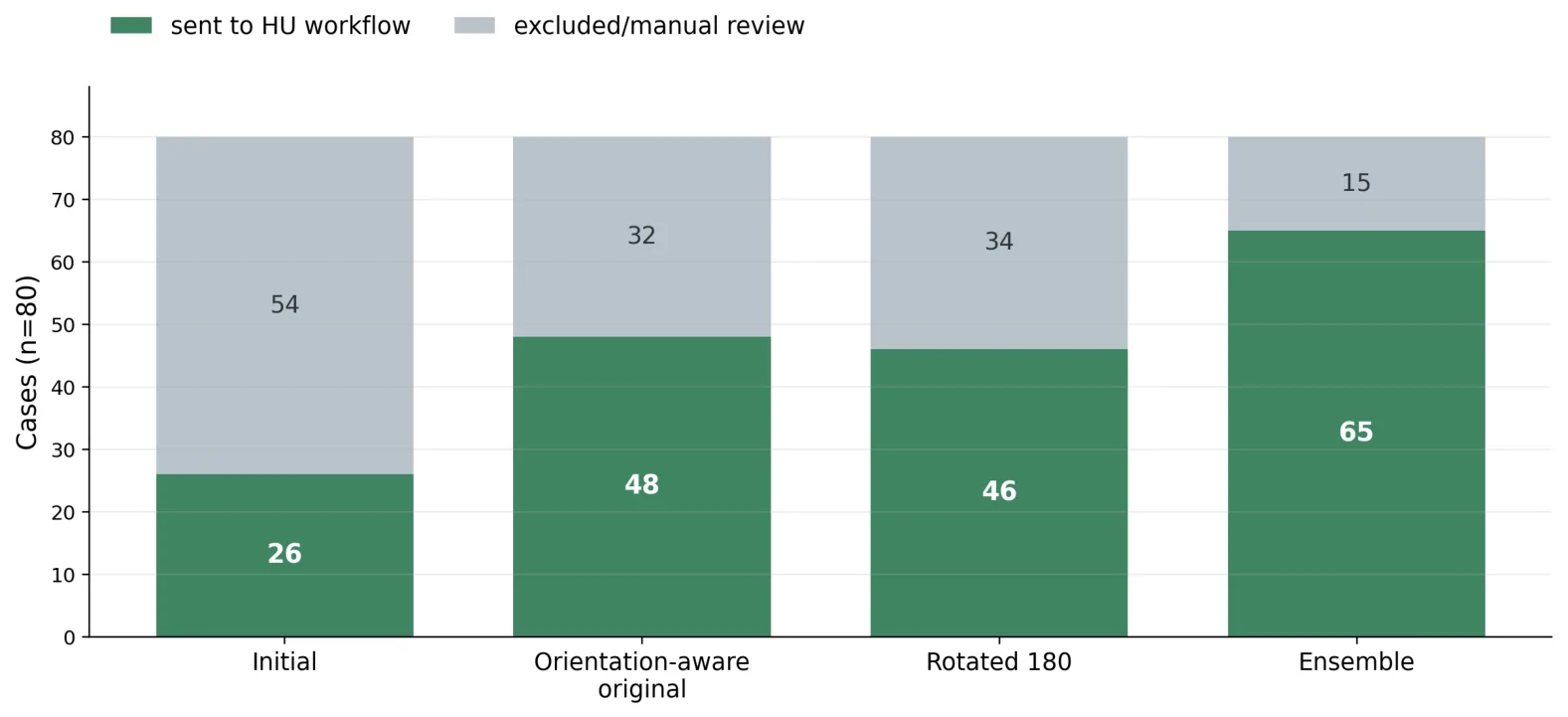
PANCREAS-CT orientation sensitivity. The orientation ensemble reduced display-orientation sensitivity by reviewing both original and 180-degree-rotated montage presentations before routing.

The Spine-Mets availability results are shown in Figure 5. The PANCREAS-CT strategy comparison and orientation analysis are shown in Figure 6 and Figure 7.

In the public Spine-Mets DICOM stress test, direct original-workflow processing produced HU outputs in 3/26 successfully downloaded CT series (11.5%; 95% CI, 4.0–29.0%), whereas the front-end-assisted route produced 21/26 outputs (80.8%; 95% CI, 62.1–91.5%). The paired discordance counts were both-success 3, front-end-only 18, baseline-only 0, and both-failure 5, giving exact McNemar p<0.001. These data support public DICOM workflow stress testing under heterogeneous metadata, scanner, and orientation conditions, not bone-disease outcome validation.

In the PANCREAS-CT non-target stress test, direct original-workflow processing produced 0/80 HU outputs (0.0%; 95% CI, 0.0–4.6%). The initial suitability-guided route sent 26/80 series to the downstream HU workflow and produced 17/80 HU outputs. Orientation ensembling routed 65/80 series and produced 43/80 HU outputs. Spine-localized FOV adaptation without explicit orientation normalization produced 53/80 HU outputs, whereas spine-localized FOV adaptation with DICOM orientation normalization produced 75/80 HU outputs (93.8%; 95% CI, 86.2–97.3%). The deterministic body-crop control produced 75/80 HU outputs, and the orientation-normalized deterministic body-crop strong-control produced 78/80 outputs (97.5%; 95% CI, 91.3–99.3%). These results indicate that DICOM orientation and geometric centering were major deployment failure modes; they also show that availability alone can overstate appropriate routing when semantic suitability and crop/segmentation quality are not considered.

Overall, the public DICOM stress tests demonstrated that heterogeneous acquisition, coverage, presentation, and orientation conditions can substantially affect downstream HU workflow availability.

### 3.3. Controls, Reviewability, and Auditable Failure Evidence

**Figure 8:**
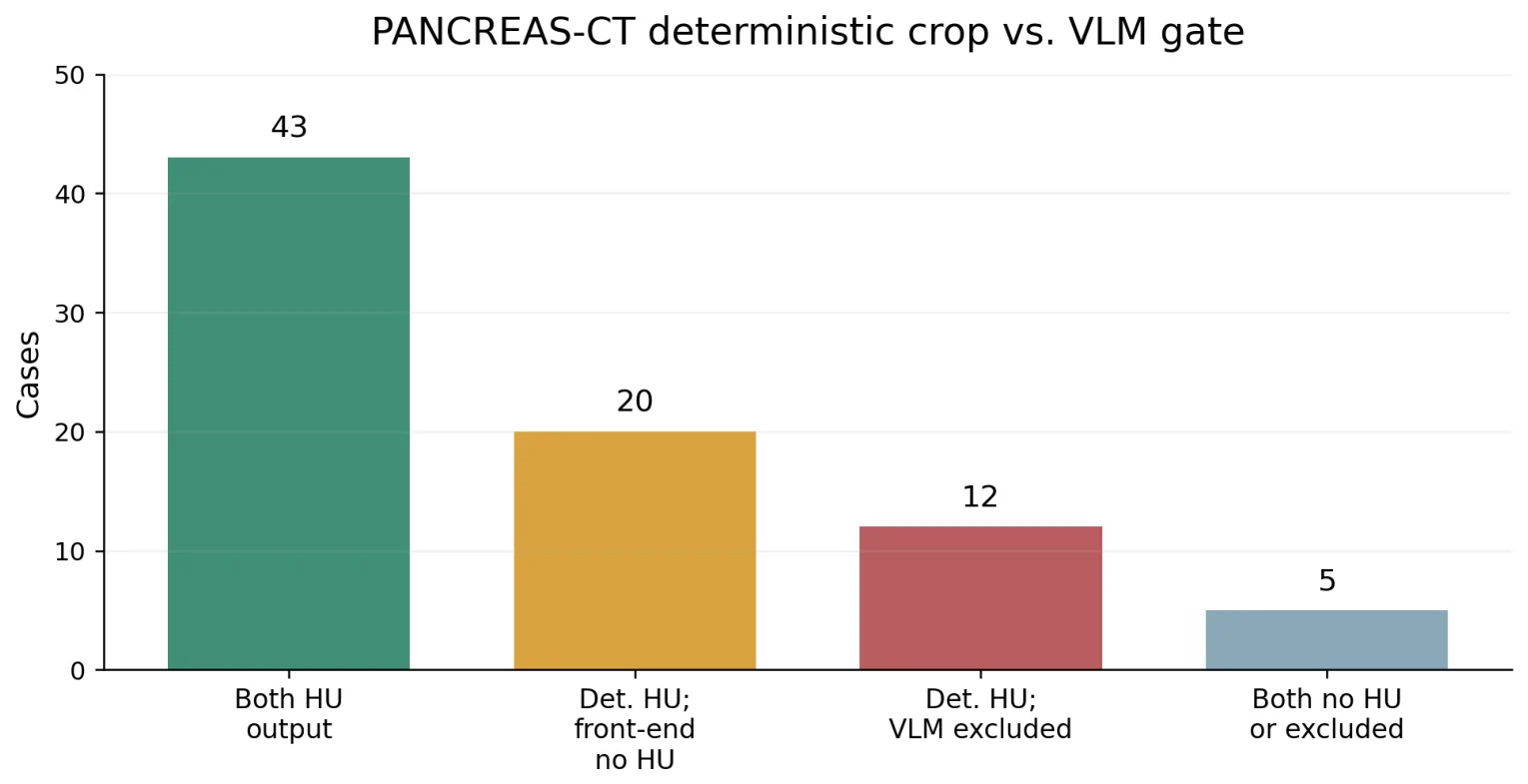
PANCREAS-CT deterministic crop versus VLM gate categories. The control highlights why numeric availability and appropriate routing must be interpreted separately.

The deterministic-crop/VLM route comparison is shown in Figure 8. Focused adjudication and route-level results are summarized in Table 2, Table 3, Table 4 and Table 5. The PANCREAS VLM-versus-deterministic contrast is provided in Supplementary Table S4. The Spine-Mets visual adjudication is provided in Supplementary Table S5, and the focused public routing review is provided in Supplementary Table S6.

Deterministic crop controls recovered many HU outputs across the pilot and public stress-test settings. In Fuji20, deterministic body crop produced 13/20 HU outputs. In PANCREAS-CT, deterministic body crop produced 75/80 HU outputs, and the orientation-normalized deterministic body-crop strong-control produced 78/80 outputs.

Among PANCREAS-CT deterministic-crop conflict outputs, 30/32 were reviewable (93.8%; 95% CI, 79.9–98.3%) and 2/32 were not reviewable (6.2%; 95% CI, 1.7–20.1%). Among VLM-excluded PANCREAS-CT cases, 12/15 exclusions were appropriate (80.0%; 95% CI, 54.8–93.0%) and 3/15 represented problematic false targeting or exclusion behavior (20.0%; 95% CI, 7.0–45.2%). Case-level contrast between orientation-ensemble VLM routing and deterministic cropping showed 43 series with HU output in both routes, 20 series with deterministic-crop HU output but no HU output after the VLM route had sent the case to the downstream workflow, 12 series with deterministic-crop HU output despite VLM exclusion, and 5 series with no output or exclusion in both routes. In Spine-Mets, 18/21 available outputs were reviewable (85.7%; 95% CI, 65.4–95.0%), 1/21 was questionable, and 7/26 total cases were not reviewable.

For the orientation-normalized deterministic body-crop strong-control, the completed non-blinded PANCREAS-CT main-adaptation-versus-strong-control visual adjudication assigned structured labels to all 80 cases. The main adaptation route was judged better in 72/80 cases and clearly better in 4/80 cases, primarily because the strong-control crop more often placed the vertebral candidate inferiorly, off center, or with less complete vertebral coverage. The strong-control was judged better in 3/80 cases because the main adaptation route likely mistargeted surrounding vertebra-like tissue or missed the vertebral target; 1/80 case was not applicable for paired comparison. The dominant strong-control issue was inferior or off-center vertebral positioning in 76/80 cases. This full visual review was interpreted as supportive reviewer evidence for centering, coverage, and reviewability rather than as a blinded statistical superiority endpoint. The full case-level comparison is provided in Supplementary Table S7, the quality summary in Supplementary Table S8, and the aggregate visual-review record in Supplementary Table S9.

Using the single-reviewer binary coverage-based suitability labels as an exploratory reference for PANCREAS-CT route-level analysis, 75/80 cases were evaluable after excluding five uncertain manual-review labels. Positive labels indicated suitable lumbar or lower-thoracolumbar coverage; negative labels indicated lumbar-insufficient or otherwise coverage-unsuitable status. The VLM routing gate had TP/FP/TN/FN = 63/0/10/2, sensitivity 96.9% (95% CI, 89.5–99.2%), specificity 100.0% (95% CI, 72.2–100.0%), accuracy 97.3%, F1 score 98.4%, false-acceptance rate 0.0%, and false-exclusion rate 3.1%. In the same evaluable set, deterministic body crop had TP/FP/TN/FN = 63/8/2/2, sensitivity 96.9%, specificity 20.0%, accuracy 86.7%, F1 score 92.6%, and false-acceptance rate 80.0%; the orientation-normalized deterministic body-crop strong-control had TP/FP/TN/FN = 64/9/1/1, sensitivity 98.5%, specificity 10.0%, accuracy 86.7%, F1 score 92.8%, and false-acceptance rate 90.0%. These exploratory metrics were calculated against single-reviewer, non-blinded anatomical-coverage labels rather than an independent clinical reference standard or complete HU-measurement suitability reference.

Together, these control and adjudication results show that numeric HU output, reviewability, appropriate exclusion, and false-target evidence must be reported as distinct deployment endpoints.

### 3.4. Representative Adaptation and Auditable Failure Evidence

**Figure 9:**
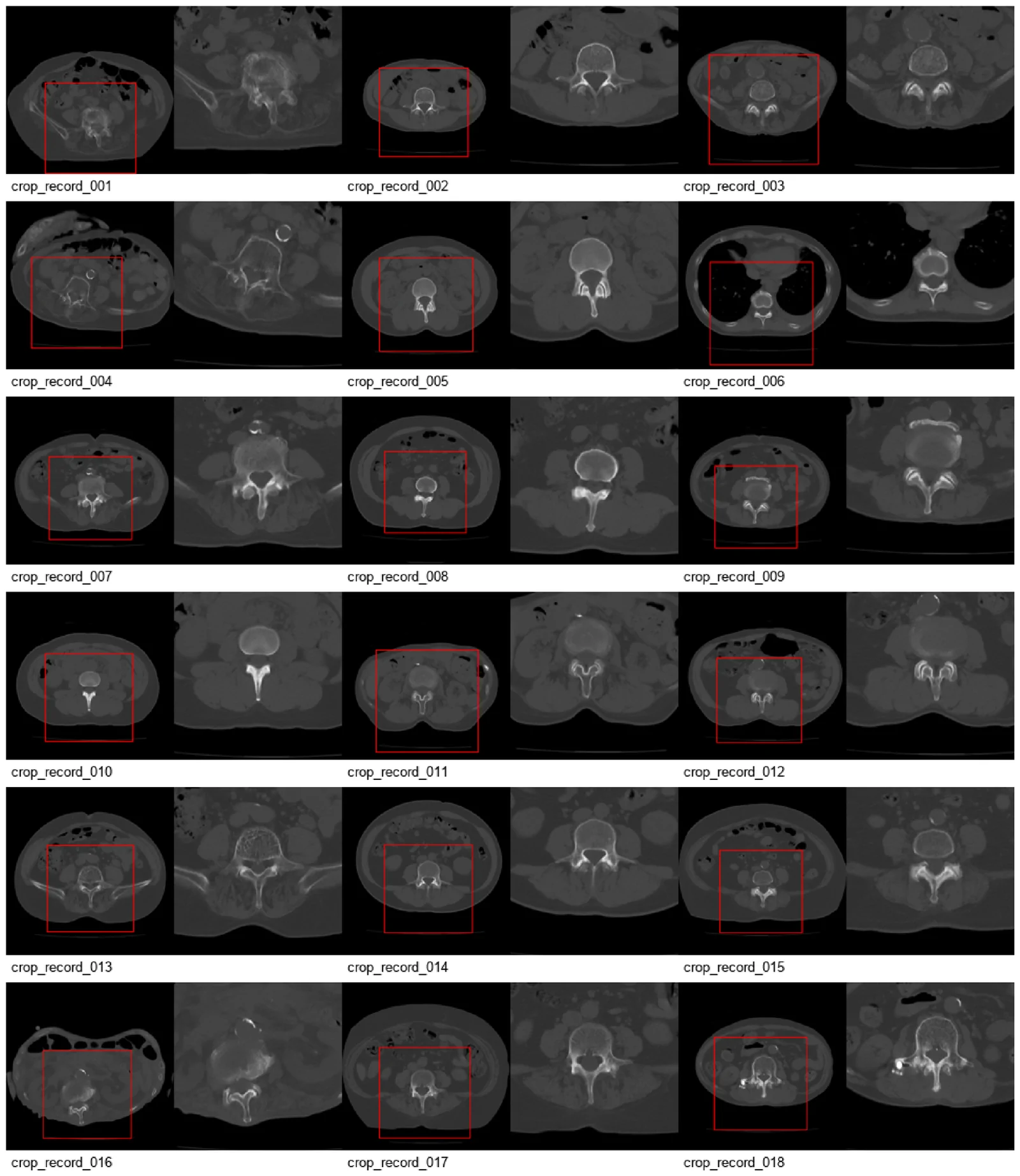
Crop previews for the 18 Fuji20 cases routed to DICOM-derived adaptation. Crop-record identifiers were assigned after routing and do not correspond directly to the original Fuji20 case identifiers. The red rectangle indicates the series-fixed physical-FOV crop used for HU-traceable DICOM-derived adaptation. Preview images are provided as audit and planning evidence rather than as final HU-calculation inputs.

**Figure 10:**
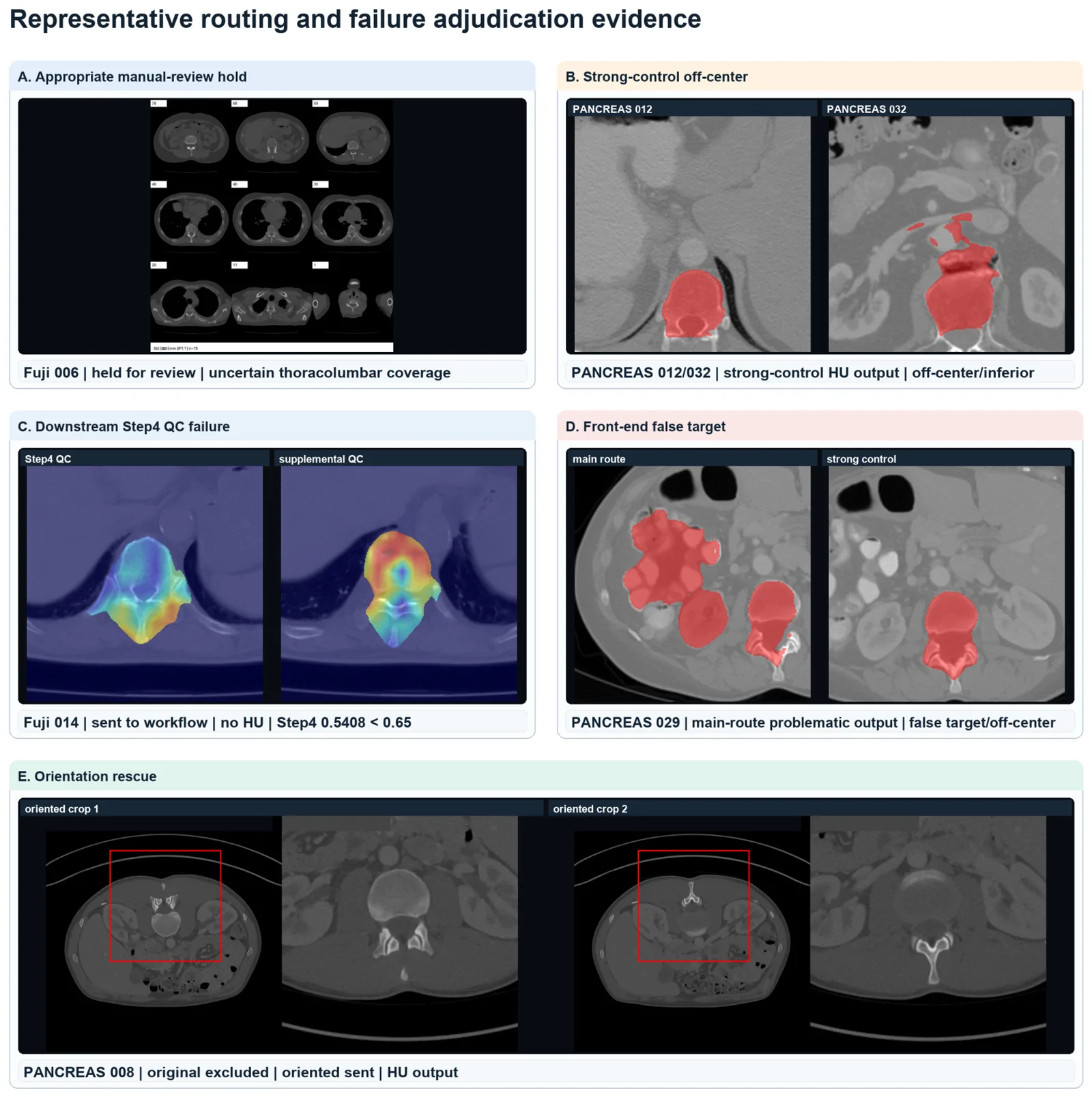
Representative routing and failure adjudication evidence. Panels illustrate manual-review hold for uncertain thoracolumbar coverage, orientation-normalized deterministic strong-control off-center targeting, downstream Step 4 quality-control failure, front-end false-target behavior, and orientation rescue. Red boxes indicate crop windows where present, red overlays indicate downstream segmentation or candidate-target evidence, and the panel title colors distinguish the illustrated evidence categories. Medical image panels were assembled from real DICOM-derived slices, crop previews, downstream QC visualizations, and downstream segmentation overlays; only display conversion, cropping, resizing, layout, and text labels were applied. Detailed case-level failure evidence is provided in Supplementary Table S3, and public routing-conflict adjudication is provided in Supplementary Tables S4–S6.

Representative adapted inputs and failure evidence are shown in Figure 9 and Figure 10.

Representative crop previews from the Fuji pilot cohort show the intended adaptation target: a series-fixed, spine-centered, bone-window-like DICOM crop suitable for downstream HU workflow inference, while final HU remains computed from DICOM-derived HU arrays rather than preview pixels.

Representative adjudication panels illustrate the kinds of real workflow evidence used to interpret routing and downstream failures: manual-review hold for uncertain thoracolumbar coverage, deterministic strong-control off-center targeting, downstream Step 4 quality-control failure, front-end false-target behavior, and orientation rescue. The panels were assembled from DICOM-derived slices and downstream workflow outputs; no generative image model was used to create, alter, or enhance the medical image content.

### 3.5. Gate-Level VLM-On/Off Routing Analysis

Gate-level routing metrics are summarized in Table 6. Detailed gate-level methods are provided in Supplementary Methods S1. Case-level gate decisions are provided in Supplementary Table S10, confusion matrices and routing metrics in Supplementary Table S11, and paired gate-decision comparisons in Supplementary Table S12. Metric plots are shown in Supplementary Figure S1, and operational consequences are shown in Supplementary Figure S2. Five cases labeled as uncertain/manual review were excluded from the binary-metric denominator, leaving 75 evaluable cases.

The gate-level VLM-on/off routing-decision analysis used the 75 evaluable PANCREAS-CT cases with binary single-reviewer suitability labels. The VLM orientation-ensemble gate had TP/FP/TN/FN = 63/0/10/2, sensitivity 96.9% (95% CI, 89.5–99.2%), specificity 100.0% (95% CI, 72.2–100.0%), accuracy 97.3%, balanced accuracy 98.5%, F1 score 98.4%, false-acceptance rate 0.0%, false-exclusion rate 3.1%, and sent 63/75 evaluable cases downstream. The no-semantic pass-through route sent all 75 evaluable cases, giving TP/FP/TN/FN = 65/10/0/0, sensitivity 100.0%, specificity 0.0%, accuracy 86.7%, balanced accuracy 50.0%, F1 score 92.9%, false-acceptance rate 100.0%, and false-exclusion rate 0.0%. The basic technical-integrity pass-through matched the no-semantic pass-through route in this PANCREAS set because all 80 series passed the basic manifest/DICOM integrity rule. The existing orientation-normalized deterministic body-crop strong-control output policy had TP/FP/TN/FN = 64/9/1/1, sensitivity 98.5%, specificity 10.0%, accuracy 86.7%, balanced accuracy 54.2%, F1 score 92.8%, false-acceptance rate 90.0%, and false-exclusion rate 1.5%.

The HU-output count shown for the no-semantic and technical-integrity pass-through policies was reused from the archived all-series orientation-normalized spine-localized route and was not generated by a fully rerun end-to-end ablation. Compared with the no-semantic pass-through route, the VLM gate reduced inappropriate routing of negative/lumbar-insufficient cases from 10/10 to 0/10, while excluding 2/65 suitable cases. Exact McNemar testing of classification correctness favored the VLM gate over the no-semantic gate (VLM-correct/no-gate-wrong = 10; no-gate-correct/VLM-wrong = 2; exact p=0.039). These results support VLM contribution primarily as suitability-aware routing rather than as HU measurement, final segmentation, ROI definition, or geometric adaptation.

### 3.6. Compatibility and Synthetic Engineering Stress

For already standardized prior-workflow-compatible selected-DICOM inputs, a bypass rule sent compatible axial inputs directly to the original HU workflow without unnecessary recropping. In the original selected-DICOM compatibility test set, the direct original HU workflow produced 43/47 HU outputs and the front-end-bypass route preserved 43/47 outputs, with 46/47 cases bypassed and no baseline-success cases lost. Among the 43 paired HU-output cases, the compatibility table used the original HU workflow output as the front-end-bypass output because the wrapper deliberately left these inputs unchanged; mean, median, and maximum absolute HU differences were therefore all 0.00 HU by design. This result supports backward compatibility and indicates that the local VLM-assisted front end did not introduce a wrapper-level change on already appropriate selected-DICOM inputs. It should not be interpreted as evidence that VLM-based preprocessing reproduces HU exactly. A controlled synthetic stress experiment created 24 variants from eight original-workflow-success cases using large-FOV padding, off-center positioning, and soft-tissue window metadata. Baseline and front-end workflows both completed 24/24 synthetic inputs. However, the front-end reduced mean absolute HU deviation from the original input for centered large-FOV variants from 3.21 to 0.97 HU and for off-center large-FOV variants from 4.19 to 0.92 HU; soft-window metadata variants remained essentially unchanged at 0.003 HU. These synthetic experiments were used as engineering robustness evidence and were not counted as additional independent clinical cases.

### 3.7. Runtime and Crop-Geometry Documentation

Existing local VLM-stage timing logs showed a median Qwen2.5-VL-7B suitability-planning latency of 3.78 s per PANCREAS-CT series in the available planning log subset (n = 10; mean 4.27 s; range 3.55–9.05 s). In the Qwen prompt-only orientation pilot using real cases, the median VLM call time was 6.96 s (n = 37; mean 7.82 s; range 3.46–21.37 s). These timings document the local VLM inference stage and should not be interpreted as a full end-to-end workflow runtime benchmark.

Existing crop-transform summaries confirmed that the intended adaptation crop was approximately 180 mm in physical width and height. Median crop width was 180.1 mm in Fuji20 main front-end records and 179.7 mm in PANCREAS spine-localized orientation-normalized, PANCREAS orientation-normalized deterministic strong-control, and Spine-Mets records. Median crop area fraction was 28.0% in Fuji20, 16.7% in both PANCREAS routes, and 19.7% in Spine-Mets. Border-touching crops occurred in 1/18 Fuji20 records and 3/26 Spine-Mets records, but in 0/80 records for each PANCREAS route. PANCREAS records underwent orientation normalization, whereas Fuji20 and Spine-Mets available records did not require this transform. These records support the interpretation that the crop was a compatibility FOV adaptation with broad candidate-anatomy coverage rather than a final vertebral or trabecular ROI; validated full-cohort target-clipping, vertebral-occupancy, or margin rates were not reported because complete reference masks were unavailable.

## 4. Discussion

The principal finding of this study is that deployment quality in automated lumbar CT HU measurement cannot be assessed by numeric HU output availability alone. In heterogeneous real-world DICOM inputs, a workflow must also determine whether a series is suitable for lumbar HU measurement, whether the target and ROI are visually reviewable, whether exclusion or manual-review hold is appropriate, and whether failure modes are auditable. The local VLM-assisted routing and HU-traceable DICOM-derived adaptation framework was therefore evaluated not simply as a way to generate more HU values, but as a deployment-quality layer upstream of an unchanged deterministic HU workflow.

The deterministic crop controls were not merely baselines; they revealed a central deployment problem. Deterministic body cropping produced 13/20 Fuji20 HU outputs and 75/80 PANCREAS-CT outputs, while the orientation-normalized deterministic body-crop strong-control produced 78/80 PANCREAS-CT outputs. These findings show that geometric FOV/orientation adaptation is powerful and can recover many numeric outputs. However, focused adjudication showed that high numeric availability can coexist with inferior vertebral centering, incomplete coverage, false targeting, or non-reviewable output. The 180 mm crop should be interpreted as a standardized spine-centered compatibility FOV, not as a tight vertebral bounding box or final trabecular ROI, and it intentionally retained surrounding anatomy to support downstream processing. In the completed non-blinded PANCREAS full visual review, the main adaptation route was judged better or clearly better than the orientation-normalized deterministic strong-control in 76/80 cases, whereas 3/80 documented remaining main-adaptation-route targeting failures and 1/80 was not applicable. This full visual review supported the qualitative advantage of main-adaptation-route centering and coverage, but it remains supportive single-reader, non-blinded reviewer evidence rather than a formally blinded superiority endpoint. Because complete reference masks were unavailable, validated full-cohort clipping, vertebral-occupancy, or margin rates were not reported.

The value of the VLM-assisted component should therefore be interpreted less as throughput improvement than as suitability routing and planning support within an integrated front-end framework. It provided series-level suitability assessment, routing decisions, exclusion/manual-review flags, and auditable planning evidence, while deterministic components performed spine-candidate localization, series-fixed FOV adaptation, and orientation normalization. The exploratory PANCREAS routing metrics were consistent with this interpretation: deterministic controls had high sensitivity and high numeric output availability, whereas the VLM routing gate reduced false acceptance against the single-reviewer coverage-based suitability labels. Because the components were combined, the present study evaluates the integrated deployment front end rather than isolating the VLM as a standalone performance source, and it does not claim VLM throughput superiority over deterministic geometric adaptation. We did not demonstrate that the VLM itself improved downstream HU accuracy; the manual HU agreement results reflect automatic-success cases processed by the unchanged downstream HU workflow after routing and adaptation. The gate-level routing-decision analysis further separates this routing role from geometric adaptation: when the comparison was limited to the source of the routing decision, the VLM gate reduced false acceptance of negative/lumbar-insufficient PANCREAS cases compared with no semantic gate or basic technical-integrity pass-through, while retaining high sensitivity. This does not constitute a fully rerun end-to-end VLM-only pipeline validation or a comparison against a trained metadata-only or montage-only classifier, but it strengthens the interpretation that the VLM contribution is semantic suitability filtering and audit-friendly planning.

The choice of a VLM should also be interpreted according to task level. Single-task localization or segmentation networks are usually faster and more computationally efficient for pixel-level tasks, and this study does not claim that a VLM should replace those models. The VLM was used for series-level multimodal suitability routing, where metadata, montage appearance, anatomical coverage, orientation, presentation state, and manual-review cues must be interpreted together before the deterministic downstream workflow is invoked.

The task boundaries of VLM-assisted routing and task-specific imaging methods are summarized in Table 7.

This perspective distinguishes deployment-quality evaluation from conventional AI performance testing. Many medical imaging AI studies emphasize segmentation accuracy, localization accuracy, or measurement agreement under curated inputs. By contrast, the present results suggest that deployment-oriented evaluation should explicitly report input suitability, reviewability, appropriate exclusion, and failure auditability alongside HU agreement or segmentation performance. This is clinically relevant because opportunistic osteoporosis screening depends on routine CT examinations that were not acquired specifically for lumbar HU measurement, and such inputs may have broad FOV, incomplete lumbar coverage, nonstandard orientation, or confusing presentation before HU is ever calculated.

The selected-DICOM compatibility analysis addresses the complementary requirement that a front end should not damage already suitable inputs. Preserving 43/47 HU outputs with no baseline-success cases lost supports a bypass route for prior-workflow-compatible selected-DICOM inputs. The 0.00 HU paired difference in 43 cases occurred by design because the wrapper deliberately preserved the original input path; it should be interpreted as wrapper non-interference rather than stochastic repeatability or evidence that VLM preprocessing reproduces HU exactly. The original workflow development/training data and the selected-DICOM test set were not used for local VLM prompt tuning, adapter optimization, or routing-threshold selection.

The public DICOM stress tests should also be interpreted according to their role. Spine-Mets was useful for scanner, protocol, disease-context, and DICOM-handling stress testing, while PANCREAS-CT exposed non-target routing, orientation, FOV, over-acceptance, and false-target scenarios. These cohorts challenge the workflow under diverse acquisition and presentation conditions, but they are not osteoporosis outcome-validation cohorts. Their value lies in showing how deployment failures appear and how an auditable workflow can separate HU availability from reviewability and appropriate exclusion.

Several limitations define the current level of evidence. Fuji20 is a small primary pilot technical cohort with limited early input-requirement inspection rather than an untouched external validation cohort. Manual ImageJ agreement was limited to 12 automatic-success paired cases and was performed by a single non-blinded physician reviewer rather than as a blinded reference-standard experiment. Reviewer adjudication included full non-blinded PANCREAS main-adaptation-versus-strong-control visual review and focused public conflict review, but it was not a blinded multi-reader reference-standard evaluation. The PANCREAS binary labels primarily captured lumbar or lower-thoracolumbar anatomical coverage, not all clinical factors that could affect HU-measurement suitability. No clinical osteoporosis outcome, fracture prediction, or diagnostic threshold classification was assessed. This study did not evaluate the diagnostic reliability of single-slice HU for osteoporosis. Single-slice HU was treated as the downstream measurement output of a previously published opportunistic assessment workflow rather than as a stand-alone diagnostic test. The framework was evaluated with one previously published downstream HU workflow and may not generalize unchanged to all HU measurement systems. The current front end does not provide definitive L1–L3 vertebral-level localization, and VLM behavior may depend on prompt wording, model version, montage construction, and runtime settings. Future work should include larger prospective multicenter and multi-vendor cohorts, external-hospital validation, expert lumbar coverage labels, blinded multi-reader manual HU references, explicit vertebral-level localization, fully rerun end-to-end matched VLM-on/off pipeline ablation, comparison with rule-based, metadata-only, montage-only, or lightweight classifier routing baselines, and direct comparison with downstream retraining or fine-tuning.

Overall, this study supports the view that deployment robustness is a distinct evaluation target for automated opportunistic CT screening systems. Suitability-aware routing, HU-traceable adaptation, deterministic downstream measurement, and auditable failure evidence are complementary to segmentation accuracy and HU agreement, and they should be considered when automated HU workflows are moved from curated data toward heterogeneous clinical DICOM environments.

## 5. Conclusions

A local VLM-assisted routing and HU-traceable DICOM-derived adaptation layer can be integrated upstream of the published auditable lumbar CT HU measurement workflow while preserving deterministic DICOM-based HU calculation and downstream quality control. In the small Fuji20 pilot cohort, the front-end-assisted route showed higher observed HU output availability than direct original-workflow processing and showed close post hoc ImageJ agreement in automatic-success cases, but no clinical osteoporosis outcome was assessed and the paired availability difference did not reach conventional statistical significance. More importantly, the control and adjudication analyses showed that numeric HU output generation alone is insufficient as evidence of deployment quality. These findings support suitability routing, reviewability assessment, appropriate exclusion, and auditable failure evidence as deployment-quality endpoints. The gate-level routing-decision analysis suggests that the VLM’s primary contribution is coverage-based suitability gating rather than HU estimation or geometric adaptation, while future work still requires larger prospective multicenter, multi-vendor, external-hospital, and multi-reader validation and a fully rerun matched pipeline ablation.

## Figures and Tables

**Table 1:** Data role summary. Each dataset was assigned a specific methodological role to avoid conflating pilot external technical evaluation, public stress testing, compatibility testing, and synthetic engineering perturbation.

Dataset	Size	Role	Primary Use	Limitations and Leakage Control
Fuji20	20 private Fuji CT cases	Primary frozen front-end pilot technical set	External HU output, ImageJ sanity check, failure evidence	Small pilot set with limited early input-requirement inspection; not definitive clinical validation or untouched external validation; not used for model training or case-level parameter fitting
Spine-Mets	26/55 public CT series downloaded	Public DICOM stress test	Scanner, protocol, and DICOM handling stress test	Spine metastasis/radiotherapy context; not an osteoporosis or bone-outcome validation set; download was limited to 26 accessible CT series
PANCREAS-CT	80 public CT series	Public non-target DICOM stress test	Routing, orientation, FOV adaptation, crop controls, over-acceptance analysis	Used for engineering stress testing and orientation strategy development; not an untouched clinical validation set
Selected-DICOM	47 compatible inputs	Wrapper non-interference control	Backward compatibility on already standardized inputs	Not used for prompt tuning, crop-adapter development, routing thresholds, or model-weight updates
Synthetic stress	24 variants from 8 original-success cases	Controlled engineering robustness test	Large-FOV, off-center, and display-window perturbations	Derived inputs only; not independent clinical cases

**Table 2:** Focused reviewer adjudication of public stress-test and routing-conflict outputs. The adjudication evaluates reviewability and appropriate exclusion.

Dataset	Review Task	Total	Appropriate Excl.	Reviewable HU	Question.	Not Reviewable/False Target
PANCREAS-CT	Deterministic-crop conflict	32	–	30	0	2
PANCREAS-CT	VLM-exclusion review	15	12	0	0	3
Spine-Mets	Public DICOM stress-test	26	–	18	1	7

**Table 3:** Route-level endpoint summary. The table places numeric availability beside reviewability, appropriate exclusion, and false-target or not-reviewable evidence to emphasize that availability alone was not treated as correctness.

Route or Evidence Set	HU Output	Reviewable Evidence	Exclusion/Failure Evidence	Role
Fuji20: direct original HU workflow	5/20	No final-route visual review	No front-end gate	Baseline throughput
Fuji20: front-end-assisted route	12/20	12 paired; ImageJ in 13/20	2 held/rejected; 5 Step 4 failures; one baseline-success case unavailable after front-end adaptation	Primary pilot route
Spine-Mets: front-end-assisted route	21/26	18 reviewable; 1 questionable	7/26 total not reviewable	Public DICOM stress test
PANCREAS-CT: orientation-ensemble VLM route	43/80	Conflict/exclusion sets reviewed	12/15 appropriate exclusions; 3 problematic	Suitability-routing evidence
PANCREAS-CT: deterministic body crop	75/80	30/32 conflict outputs reviewable	2/32 conflict outputs not reviewable	Geometric control
PANCREAS-CT: orientation-normalized deterministic crop	78/80	Full 80-case visual comparison	Inferior/off-center position in 76/80; strong-control favored in 3/80	Strong geometric control
Selected-DICOM bypass route	43/47 preserved	43 paired outputs unchanged by design	No baseline-success case lost	Wrapper non-interference

**Table 4:** Exploratory PANCREAS-CT route-level metrics against single-reviewer, non-blinded binary suitability labels. Five cases labeled as uncertain/manual review were excluded from the binary-metric denominator, leaving 75 evaluable cases.

Route	TP/FP/TN/FN	Sensitivity	Specificity	Accuracy	PPV/NPV	F1	False Accept.	False Excl.
VLM routing gate	63/0/10/2	96.9% (89.5–99.2)	100.0% (72.2–100.0)	97.3%	100.0/83.3%	98.4%	0.0%	3.1%
Deterministic body crop	63/8/2/2	96.9% (89.5–99.2)	20.0% (5.7–51.0)	86.7%	88.7/50.0%	92.6%	80.0%	3.1%
Orientation-normalized deterministic crop	64/9/1/1	98.5% (91.8–99.7)	10.0% (1.8–40.4)	86.7%	87.7/50.0%	92.8%	90.0%	1.5%

**Table 5:** PANCREAS-CT full non-blinded main-adaptation-versus-strong-control visual adjudication comparing the main adaptation route with the orientation-normalized deterministic body-crop strong control.

Quality Comparison	Dominant Issue	n	Short Interpretation
Main adaptation route better	Strong-control off-center/inferior vertebral position	72	Main adaptation route more centered or complete
Main adaptation route clearly better	Strong-control off-center/inferior vertebral position	4	Stronger main-adaptation-route visual advantage
Strong-control better	No dominant strong-control issue	3	Main-route false-target or missed-target failure
Not applicable	No paired visual comparison	1	No paired quality comparison

**Table 6:** Gate-level VLM-on/off routing-decision analysis against single-reviewer PANCREAS-CT suitability labels. The basic technical-integrity pass-through used only minimal manifest/DICOM integrity fields and did not use downstream outcome or reviewer labels.

Route	Sensitivity (95% CI)	Specificity (95% CI)	Accuracy	Balanced Acc.	False Acceptance	False Exclusion	Sent Among Evaluable Cases (n = 75)
VLM gate	96.9% (89.5–99.2%)	100.0% (72.2–100.0%)	97.3%	98.5%	0.0%	3.1%	84.0%
No semantic pass-through	100.0% (94.4–100.0%)	0.0% (0.0–27.8%)	86.7%	50.0%	100.0%	0.0%	100.0%
Technical-integrity pass-through	100.0% (94.4–100.0%)	0.0% (0.0–27.8%)	86.7%	50.0%	100.0%	0.0%	100.0%

Note: The full-cohort VLM orientation-ensemble route sent 65/80 series. The percentages shown in this table are restricted to the 75 cases included in the binary gate-level analysis.

**Table 7:** Task-boundary comparison between VLM-assisted routing and task-specific imaging methods. This table summarizes task boundaries and does not constitute a direct performance or computational benchmark.

Method Category	Primary Input	Primary Output	Pixel-Level Task	Series-Level Routing	Uses DICOM Metadata	Role in Current Study
U-Net/nnU-Net	Image arrays	Segmentation mask	Yes	No	Usually no	Reference class for pixel segmentation
TotalSegmentator	CT volume	Organ/bone masks	Yes	Limited	Usually no	Reference class for anatomy segmentation
Rule-based router	DICOM tags	Eligibility rule	No	Yes	Yes	Nonsemantic routing comparator concept
VLM-assisted routing	Metadata + montage	Suitability/ planning flags	No	Yes	Yes	Front-end planning component
Downstream HU workflow	DICOM-derived HU array	ROI and HU summary	Yes	After input gate	Yes	Measurement authority

## Data Availability

The original published HU workflow code, inference pipeline, example artifacts, and usage instructions are publicly available at https://github.com/WEISHANGAZHE/automated-single-slice-lumbar-qct-hu (accessed on 1 August 2026). De-identified case-level intermediate tables, prompts, routing records, and evidence figures supporting the reported analyses are provided in the Supplementary Materials. Raw private clinical imaging data cannot be publicly released because of institutional and ethical restrictions. Public DICOM stress-test data were obtained from TCIA collections.

## Supplementary Materials

The following supporting information can be downloaded at: https://www.mdpi.com/article/10.3390/jimaging12080385/s1. The supplementary tables are organized into four groups: Fuji20 pilot evidence (Tables S1–S3), public DICOM stress-test adjudication (Tables S4–S6), PANCREAS main-adaptation-versus-strong-control visual comparison evidence (Tables S7–S9), and gate-level routing-decision analysis (Supplementary Methods S1, Tables S10–S12, and Figures S1 and S2). Supplementary Table S1, Fuji20 case-level baseline, deterministic crop, front-end, and manual HU outcomes; Supplementary Table S2, Fuji20 manual ImageJ slice and ROI review mapping; Supplementary Table S3, Fuji20 case-level failure evidence with shortened labels; Supplementary Table S4, PANCREAS-CT VLM-versus-deterministic crop contrast summary; Supplementary Table S5, Spine-Mets public DICOM stress-test visual adjudication summary; Supplementary Table S6, Focused public routing and visual adjudication summary; Supplementary Table S7, PANCREAS-CT full main-adaptation-versus-strong-control visual adjudication summary; Supplementary Table S8, Main-versus-strong-control quality summary; Supplementary Table S9, Aggregate reviewer visual review record for the PANCREAS-CT paired main-adaptation-route versus strong-control comparison; Supplementary Methods S1, Gate-level VLM-on/off routing analysis and label provenance; Supplementary Table S10, PANCREAS-CT case-level gate decisions and coverage-based suitability labels; Supplementary Table S11, Gate-level confusion matrices and routing metrics with 95% confidence intervals; Supplementary Table S12, Paired gate-decision comparisons and exact McNemar tests; Supplementary Figure S1, Gate-level routing metrics with 95% confidence intervals; Supplementary Figure S2, Operational consequences of PANCREAS-CT routing policies; and Supplementary Technical Appendix, prompt and JSON schema summary, adapter details, failure review checklist, endpoint confidence intervals, exploratory route-level metric definitions, runtime and software environment notes, crop/FOV geometry summary, and public data access notes.

## Abbreviations

The following abbreviations are used in this manuscript:

CT	Computed tomography
DICOM	Digital Imaging and Communications in Medicine
FOV	Field of view
HU	Hounsfield unit
ICC	Intraclass correlation coefficient
LoRA	Low-rank adaptation
MAE	Mean absolute error
PACS	Picture Archiving and Communication System
PPV	Positive predictive value
NPV	Negative predictive value
QCT	Quantitative computed tomography
ROI	Region of interest
RMSE	Root mean square error
TCIA	The Cancer Imaging Archive
VLM	Vision-language model
