# Supplementary material for: VLM-Assisted Routing and HU-Traceable DICOM Adaptation for Lumbar CT HU Measurement: A Deployment-Oriented Pilot Technical Evaluation
Source: J Imaging. 2026 Aug 14;12(8):385. doi: 10.3390/jimaging12080385 (PMC13515003; doi:10.3390/jimaging12080385)
Supplement: Supplementary file 1 [file jimaging-12-00385-s001.zip › supplementary_materials_proof_corrected_final.pdf]

Supplementary Materials: VLM-Assisted Routing and HU-Traceable DICOM Adaptation for Lumbar CT HU Measurement: A Deployment-Oriented Pilot Technical Evaluation

Zhe-Yu Ye [1], Jun-Mu Peng [1] and Tamotsu Kamishima [1,*]

Supplementary Tables

The supplementary tables are organized into four groups: Fuji20 pilot evidence (Tables S1–S3), public DICOM stress-test adjudication (Tables S4–S6), PANCREAS main-adaptation-versus-strong-control visual comparison evidence (Tables S7–S9), and gate-level routing-decision analysis (Supplementary Methods S1, Tables S10–S12, and Figures S1–S2). Machine-readable CSV files are provided separately with filenames matching the corresponding supplementary table numbers.

Table S1. Fuji20 case-level baseline, deterministic crop, front-end, and manual HU outcomes. HU values are shown only when available.

No.	Original case ID	Base	Base HU	Crop	Crop HU	Front	Front HU	Manual HU
001	DICOMSAVE-20260425161526-000	No HU		No HU		No HU		174.6
002	DICOMSAVE-20260425161945-000	No HU		HU output	182.2	HU output	182.1	178.6
003	DICOMSAVE-20260425162331-000	No HU		HU output	149.1	HU output	148.9	152.3
004	DICOMSAVE-20260425162718-000	No HU		No HU		No HU		
005	DICOMSAVE-20260425162928-000	HU output	240.3	HU output	256.9	HU output	258.2	267.0
006	DICOMSAVE-20260425163254-000	HU output	132.7	No HU		not run manual review or rejected		
007	DICOMSAVE-20260425170718-000	No HU		HU output	83.5	HU output	89.1	84.6
008	DICOMSAVE-20260507060622-000	No HU		HU output	153.3	HU output	155.7	150.8
009	DICOMSAVE-20260507061216-000	No HU		No HU		No HU		
010	DICOMSAVE-20260507061310-000	No HU		HU output	74.1	HU output	73.8	68.7
011	DICOMSAVE-20260507062310-000	No HU		No HU		not run manual review or rejected		
012	DICOMSAVE-20260507062343-000	No HU		HU output	222.3	HU output	226.0	215.0
013	DICOMSAVE-20260507064113-000	HU output	-2.4	No HU		No HU		
014	DICOMSAVE-20260507072231-000	No HU		No HU		No HU		
015	DICOMSAVE-20260507073237-000	HU output	28.4	HU output	22.4	HU output	22.9	25.1
016	DICOMSAVE-20260507073715-000	No HU		HU output	141.0	HU output	132.7	131.9
017	DICOMSAVE-20260507081751-000	No HU		HU output	85.6	HU output	84.7	80.0
018	DICOMSAVE-20260507083228-000	No HU		HU output	65.3	No HU		
019	DICOMSAVE-20260507084420-000	No HU		HU output	72.9	HU output	68.6	65.9
020	DICOMSAVE-20260507085559-000	HU output	56.6	HU output	60.9	HU output	60.2	64.1

Table S2. Fuji20 manual ImageJ slice and ROI review mapping. Blank or unavailable values are displayed as dashes.

Folder	Case	Auto HU	Auto slices	Manual HU	Manual slices	Slice review	ROI review
001	001	–	–	174.6	64;67;71;73;74	Consistent	Accepted
002	002	182.1	64;67;71;73;74;80	178.6	64;67;71;73;80	Accepted	Accepted
003	003	148.9	79	152.3	72;79	Accepted	Accepted
004	004	–	–	–	–	–	–
005	005	258.2	61;62;63;67;69;75	267.0	61;63;67;69;75;76	Accepted	Accepted
006	006	–	–	–	–	–	–
007	007	89.1	67;73;79;80;86;87	84.6	67;73;79;80;86;87	Accepted	Accepted
008	008	155.7	65;67;72;73;78;79	150.8	65;67;72;73;78;79	Accepted	Accepted
009	009	–	–	–	–	–	–
010	010	73.8	61;73;74;80;81;89	68.7	73;74;80;81	Accepted	Accepted
011	011	–	–	–	–	–	–
012	012	226.0	71;77;78	215.0	71;77;78	Accepted	Accepted
013	013	–	–	–	–	–	–
014	014	–	–	–	–	–	–
015	015	22.9	64;69;76	25.1	64;68;69	Accepted	Accepted
016	016	132.7	60;67;69;74;75;76	131.9	60;67;69;74;75;76	Accepted	Accepted
017	017	84.7	60;67;68;73	80.0	60;67;68;73	Accepted	Accepted
018	018	–	–	–	–	–	–
019	019	68.6	67;68;69;74;76	65.9	67;68;69;74;76	Accepted	Accepted
020	020	60.2	73;86	64.1	73	Accepted	Accepted

Table S3. Fuji20 case-level failure evidence with shortened labels. Detailed logs were retained locally for audit.

Case	Baseline	Front-end	Baseline Step 4	Front-end Step 4	Category
001	No HU	No HU	0.7346	0.6364	Step 4 fail
002	No HU	HU output	0.9320	0.9302	Rescued
003	No HU	HU output	0.6471	0.7797	Rescued
004	No HU	No HU	0.6815	0.6127	Step 4 fail
005	HU output	HU output	0.8437	0.8703	both workflows completed
006	HU output	not run manual review or rejected	0.9230	–	front end manual review or rejection
007	No HU	HU output	0.9652	0.9579	Rescued
008	No HU	HU output	–	0.9330	Rescued
009	No HU	No HU	0.0975	0.0379	Step 4 fail
010	No HU	HU output	0.9664	0.9631	Rescued
011	No HU	not run manual review or rejected	0.2087	–	front end manual review or rejection
012	No HU	HU output	0.7261	0.8402	Rescued
013	HU output	No HU	0.8851	0.6458	lost after front end
014	No HU	No HU	0.8694	0.5408	Step 4 fail
015	HU output	HU output	0.8145	0.9117	both workflows completed
016	No HU	HU output	0.9430	0.9797	Rescued
017	No HU	HU output	0.8324	0.8808	Rescued
018	No HU	No HU	0.7518	0.4743	Step 4 fail
019	No HU	HU output	0.9248	0.9531	Rescued
020	HU output	HU output	0.8086	0.8403	both workflows completed

Table S4. PANCREAS-CT VLM-versus-deterministic crop contrast summary.

Contrast category	n	Interpretation
Both routes produced HU	43	Availability concordant; not evidence of VLM-exclusive benefit.
Crop output, front-end sent but no HU	20	Geometric crop recovered outputs that the sent front-end route did not complete.
Crop output despite VLM exclusion	12	Key conflict set for appropriate-exclusion review.
No output or excluded in both routes	5	Neither route provided a reviewable numeric endpoint.

Table S5. Spine-Mets public DICOM stress-test visual adjudication summary.

Visual category	n	Interpretation
Reviewable HU output	18	Available output judged visually reviewable.
Questionable output	1	Available output required caution.
Not reviewable	7	No reviewable output or unsuitable visual evidence.

Table S6. Focused public routing and visual adjudication summary.

Dataset	Review task	Total	Appropriate exclusion	Reviewable HU	Questionable	Not reviewable / false targeting
PANCREAS-CT	deterministic-crop conflict	32	–	30	0	2
PANCREAS-CT	VLM-exclusion review	15	12	–	0	3
Spine-Mets	public DICOM stress-test	26	–	18	1	7

Table S7. PANCREAS-CT full main-adaptation-versus-strong-control visual adjudication summary.

Quality comparison	Dominant strong-control issue	n	Interpretation
Main adaptation route better	Off-center/inferior vertebral position	72	Main adaptation route provided more centered or complete vertebral coverage in most cases.
Main adaptation route clearly better	Off-center/inferior vertebral position	4	Stronger examples where strong-control positioning was visually inferior.
Strong control better	None assigned	3	Main adaptation route likely false-targeted surrounding vertebra-like tissue or missed the vertebral target.
Not applicable	None assigned	1	No meaningful paired visual comparison was assigned.

Table S8. Main-versus-strong-control quality summary.

Quality comparison	Dominant strong-control issue	n	Interpretation
Main adaptation route better	Strong control off-center/inferior vertebral position	72	Supports more stable main-adaptation-route vertebral centering and coverage in the full visual review.
Main adaptation route clearly better	Strong control off-center/inferior vertebral position	4	Highlights stronger examples of main-adaptation-route crop/segmentation/ROI quality advantage.
Strong control better	No dominant strong-control issue	3	Documents remaining main-adaptation-route false-target or missed-target failure.
Not applicable	No paired visual comparison	1	No meaningful paired quality comparison was assigned.

Table S9. Aggregate reviewer visual review record for the PANCREAS-CT paired main-adaptation-route versus strong-control comparison.

Review scope	Evidence type	Total	Main adaptation route favored	Strong control favored	N/A	Dominant visual mechanism
PANCREAS full paired visual review	Non-blinded aggregate reviewer record	80	76 (72 better; 4 clearly better)	3	1	Strong control often placed the candidate low, off center, or with partial coverage.

Supplementary Methods S1. Gate-Level VLM-On/Off Routing Analysis and Label Provenance

The gate-level VLM-on/off routing-decision analysis was performed to evaluate the semantic routing decision separately from downstream geometric adaptation. The VLM-on decision was the archived VLM orientation-ensemble gate. The no-semantic pass-through decision sent all technically eligible PANCREAS-CT series at the routing-decision level. The basic technical-integrity pass-through used only minimal manifest and DICOM integrity fields and did not constitute a semantic or anatomical suitability router. Deterministic body-crop and orientation-normalized deterministic strong-control policies were included as high-sensitivity geometric output policies rather than pure routing-only gates. Five cases

labeled as uncertain/manual review were excluded from the binary-metric denominator, leaving 75 evaluable cases, and were retained separately in operational counts. The binary labels should be interpreted as coverage-based routing labels rather than complete clinical HU-measurement suitability labels.

Table S10. PANCREAS-CT case-level gate decisions and coverage-based suitability labels. The complete machine-readable table is provided as the S10 CSV file.

File	Denominator	Content
S10 CSV	80 PANCREAS-CT series	Case-level reviewer coverage-based suitability label, uncertainty flag, VLM gate decision, no-semantic pass-through decision, basic technical-integrity pass-through decision, deterministic output-policy status, strong-control output-policy status, and archived route/control output provenance.

Table S11. Gate-level confusion matrices and routing metrics with 95% confidence intervals. Complete machine-readable tables are provided as the S11 CSV files.

Route/policy	TP	FP	TN	FN
VLM gate	63	0	10	2
No semantic pass-through	65	10	0	0
Basic technical-integrity pass-through	65	10	0	0
Body-crop output policy	63	8	2	2
Strong-control output policy	64	9	1	1

Table S12. Paired gate-decision comparisons and exact McNemar tests. The complete machine-readable table is provided as the S12 CSV file.

Comparison	Decision discordance p	Correctness discordance p	Denominator
VLM gate vs no semantic pass-through	0.00049	0.039	75 evaluable cases
VLM gate vs basic technical-integrity pass-through	0.00049	0.039	75 evaluable cases
VLM gate vs deterministic body-crop output policy	0.039	0.039	75 evaluable cases
VLM gate vs orientation-normalized deterministic strong-control output policy	0.0020	0.021	75 evaluable cases

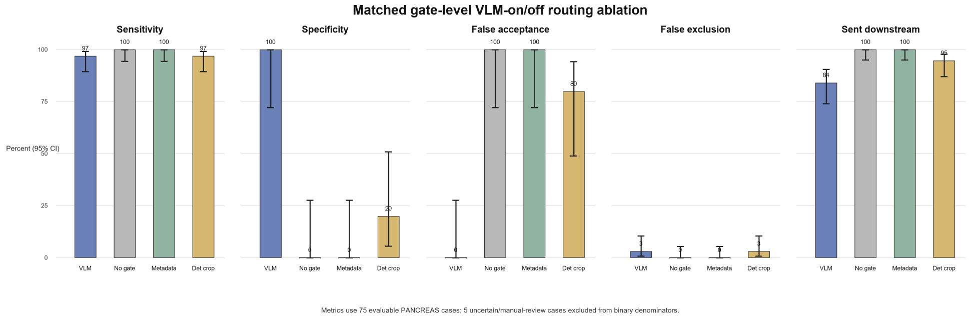

Figure S1. Gate-level routing metrics with 95% confidence intervals. Metrics were calculated against single-reader, non-blinded binary PANCREAS-CT coverage-based suitability labels; five cases labeled as uncertain/manual review were excluded from the binary-metric denominator, leaving 75 evaluable cases. Deterministic crop policies are shown as existing geometric output controls rather than pure routing-only gates.

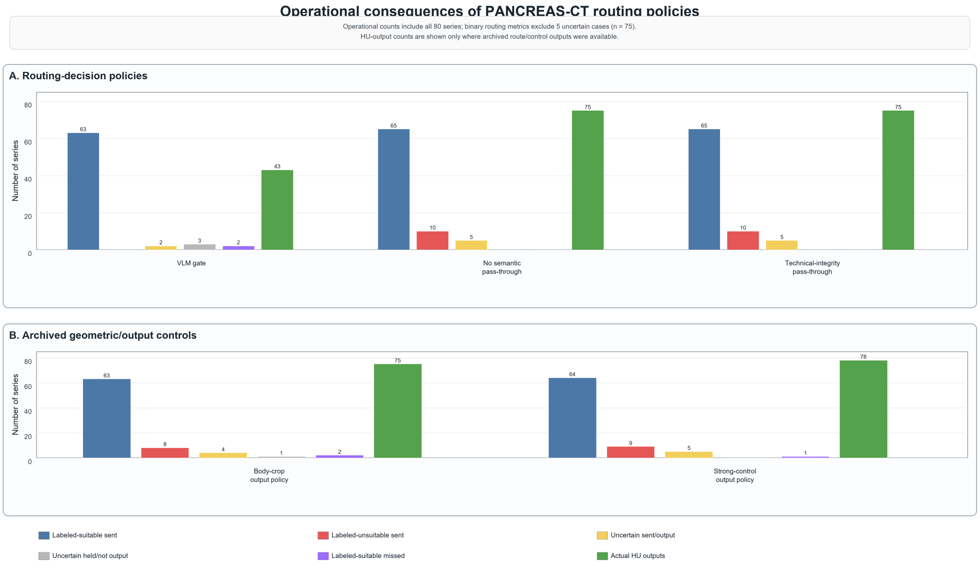

Figure S2. Operational consequences of PANCREAS-CT routing policies. Operational counts include all 80 PANCREAS-CT series, whereas binary routing metrics exclude five uncertain cases and use n = 75. HU-output counts are shown only where archived route/control outputs were available and should not be interpreted as fully rerun end-to-end matched ablation results.

Supplementary Technical Appendix

Prompt and JSON Schema Summary

The local VLM prompt instructed the model to act as a CT input planner and suitability reviewer only. It was explicitly forbidden from measuring HU, issuing diagnoses, replacing segmentation, or defining the final ROI. The evaluated Qwen2.5-VL-7B model was used as an off-the-shelf local Ollama package (`qwen2.5vl:7b`); no task-specific VLM weight training, fine-tuning, low-rank adaptation (LoRA) tuning, or case-level optimization was performed. At the post-submission environment audit, the local Ollama installation was version 0.31.2 and reported the `qwen2.5vl:7b` package as model ID `5ced39dfa4ba`, architecture label corresponding to Qwen2.5-VL, 8.3B parameters, and `Q4_K_M` quantization. The model was run locally through a JSON-response inference interface using script-level temperature 0.0 and a 512-token response limit. Each request included DICOM metadata and a 3×3 montage generated from sampled axial slices. The required JSON fields were grouped as follows:

- Modality and orientation: axial-CT status, spine-related status, and body-region text.
- Lumbar coverage and exclusion flags: lumbar/L1–L3 candidate status and thoracic-dominant or lumbar-insufficient coverage status.
- Presentation and geometry: recommended window width/level, target physical FOV, and crop strategy.
- Workflow routing: routing decision, quality flag, manual-review flag, confidence, and short notes.

The VLM output was used for planning and routing only. The semantic routing labels were "send to downstream HU workflow", "manual review", and "unsuitable input". These labels controlled whether HU-traceable DICOM-derived adaptation was attempted; they did not define the final ROI or HU value.

Adapter Details

Montages were created for review and audit only. Preview windowing used a bone-window-like display and did not alter the source DICOM values used for final

HU calculation. Accepted or reviewable DICOM series were adapted by deterministic series-fixed physical-FOV cropping. The default target FOV was 180 mm, converted to pixels using PixelSpacing with minimum and maximum crop-size guards. For long acquisitions, the sampled range emphasized the lower thoracolumbar and lumbar candidate region. The crop center was estimated from sampled slices and then applied consistently across the selected series range rather than being reselected slice by slice. DICOM orientation normalization used ImageOrientationPatient and an image-content check to determine whether a 180-degree rotation was required for prior-workflow-compatible axial presentation. Source DICOM files were not overwritten.

For derivative handling, the adapted files were saved as internal inference copies rather than clinical DICOM objects for PACS interchange. RescaleSlope, RescaleIntercept, PixelSpacing, SliceThickness, and acquisition metadata were retained. Rows, Columns, PixelData, WindowCenter, WindowWidth, SmallestImagePixelValue, LargestImagePixelValue, and ImageComments were updated. StudyInstanceUID, SeriesInstanceUID, SOPInstanceUID, ImageOrientationPatient, and ImagePositionPatient were retained and were not regenerated or recalculated in the research derivative. A separate transform manifest stored the source file path, selected series UID, crop box, adapted output path, and orientation operation. This was acceptable for this study because the downstream workflow performs image-coordinate segmentation and ROI construction on stored pixels and uses DICOM-derived HU values, not patient-coordinate spatial measurement. The derivative copies should therefore not be interpreted as standards-complete derived DICOM images for clinical exchange; such an implementation should generate derivative UIDs and update patient-coordinate geometry consistently with the DICOM standard. Final HU values were never calculated from PNG previews, montage images, normalized images, or VLM outputs.

Failure Review Checklist

Visual adjudication used a checklist covering lumbar or lower-thoracolumbar candidate visibility, crop acceptability, ROI usability, HU-output reviewability, failure appropriateness, final visual acceptability, and free-text reason. Common reason labels included confirmed non-lumbar or lumbar-insufficient exclusion, off-center or false spine target, poor bone quality, severe artifact, and downstream quality-control failure. The checklist was used to separate numeric availability from reviewable output and appropriate exclusion.

Endpoint Confidence Intervals

Wilson 95% confidence intervals were computed for key binary availability and adjudication endpoints using the submitted case-level tables. Fuji20 direct original HU availability was 5/20 (25.0%; 95% CI, 11.2–46.9%), front-end-assisted HU availability was 12/20 (60.0%; 95% CI, 38.7–78.1%), and deterministic body-crop HU availability was 13/20 (65.0%; 95% CI, 43.3–81.9%). Spine-Mets front-end HU availability was 21/26 (80.8%; 95% CI, 62.1–91.5%). PANCREAS-CT spine-localized FOV adaptation with orientation normalization produced 75/80 HU outputs (93.8%; 95% CI, 86.2–97.3%), whereas the orientation-normalized deterministic body-crop strong control produced 78/80 outputs (97.5%; 95% CI, 91.3–99.3%).

For visual adjudication endpoints, PANCREAS-CT deterministic-crop conflict outputs were reviewable in 30/32 cases (93.8%; 95% CI, 79.9–98.3%), and VLM-exclusion review found 12/15 appropriate exclusions (80.0%; 95% CI, 54.8–93.0%) with 3/15 problematic or not-reviewable exclusions (20.0%; 95% CI, 7.0–45.2%). Spine-Mets reviewability among available outputs was 18/21 (85.7%; 95% CI, 65.4–95.0%). In the completed non-blinded

PANCREAS full visual review, the main adaptation route was judged better or clearly better than the orientation-normalized deterministic strong control in 76/80 cases (95.0%; 95% CI, 87.8–98.0%), the strong control was judged better in 3/80 cases (3.8%; 95% CI, 1.3–10.5%), and 1/80 case was not applicable (1.2%; 95% CI, 0.2–6.7%). The dominant strong-control issue was inferior or off-center vertebral positioning in 76/80 cases (95.0%; 95% CI, 87.8–98.0%). Exploratory route-level sensitivity and specificity were computed only against the single-reviewer binary suitability labels. These intervals and metrics describe technical and adjudication proportions, not diagnostic accuracy or osteoporosis outcome performance.

Exploratory Route-Level Metric Definitions

Exploratory PANCREAS-CT routing metrics were reconstructed from case-level reviewer labels and route-output tables. Positive labels were cases with reviewer suitability beginning with "suitable" (suitable lumbar or lower thoracolumbar coverage). Negative labels were cases with reviewer suitability beginning with "unsuitable" (lumbar-insufficient or otherwise coverage-unsuitable for the lumbar HU workflow). Five cases labeled as uncertain/manual review were excluded from the binary-metric denominator, leaving 75 evaluable cases. These labels primarily captured anatomical coverage and did not constitute a complete clinical HU-measurement suitability reference covering all acquisition, artifact, lesion, fracture, or diagnostic-threshold considerations. The reviewer context was single-reader, non-blinded, and post-hoc using predefined workflow evidence; these metrics are therefore not an independent blinded multi-reader diagnostic performance evaluation.

Route-level metric summary.

Complete route-level metric intervals are provided in the S11 CSV files and summarized in Supplementary Table S11 above; no additional numbered table is introduced here.

Runtime and Software Environment Notes

The local VLM component was run through a local JSON-response inference interface using the off-the-shelf `qwen2.5vl:7b` Ollama package, script-level temperature 0.0, and a 512-token response limit. The workstation GPU was an NVIDIA GeForce RTX 4090D with 24564 MiB total VRAM and driver version 596.36 at the time of post-submission environment audit. Existing VLM timing logs recorded PANCREAS-CT VLM suitability planning for 10 records (median 3.78 s, mean 4.27 s per request) and a Qwen prompt-only orientation pilot in 37 real-case records (median 6.97 s, mean 7.82 s per request). These timings are reported as existing-log evidence for the local VLM calls and should not be interpreted as a complete end-to-end runtime benchmark of the full downstream HU workflow.

Crop/FOV Geometry Summary

Existing adapter transform manifests were audited to quantify the FOV adaptation geometry. The Fuji20 main front-end adapter had 18 available transform records with median crop width/height of approximately 180.1/180.1 mm, median crop area fraction 28.0%, median crop-center x/y fractions 0.49/0.64, and 1/18 border-touching crops. The Spine-Mets main front-end adapter had 26 transform records with median crop width/height of approximately 179.7/179.7 mm, median crop area fraction 19.7%, median crop-center x/y fractions 0.50/0.61, and 3/26 border-touching crops. The PANCREAS spine-localized orientation-normalized adapter had 80 transform records with median crop width/height of approximately 179.7/179.7 mm, median crop area fraction 16.7%, median

crop-center x/y fractions 0.51/0.37, 0/80 border-touching crops, and 80/80 records with 180-degree rotation normalization. The PANCREAS orientation-normalized deterministic body-crop strong control had 80 transform records with median crop width/height of approximately 179.7/179.7 mm, median crop area fraction 16.7%, median crop-center x/y fractions 0.50/0.51, 0/80 border-touching crops, and 80/80 records with 180-degree rotation normalization. These summaries support the interpretation that the adapted FOV is a high-coverage input presentation for downstream processing, not the final trabecular ROI; final ROI geometry remains determined by the downstream HU workflow. Full-cohort vertebral mask occupancy, target clipping, and background fraction were not claimed because complete validated masks were unavailable.

Public Data Access Notes

Spine-Mets-CT-SEG metadata listed 55 CT series. Twenty-six were successfully downloaded and extracted during this technical evaluation; 29 were retained as download/interface failures. PANCREAS-CT was downloaded as 80 DICOM series and used as a public non-target DICOM stress-test cohort. These public cohorts were used for deployment-oriented stress testing and ablation evidence, not for clinical bone-disease outcome evaluation.
